# Semi-permeable species boundaries in Iberian barbels (*Barbus* and *Luciobarbus*, Cyprinidae)

**DOI:** 10.1186/s12862-015-0392-3

**Published:** 2015-06-12

**Authors:** Hugo F Gante, Ignacio Doadrio, Maria Judite Alves, Thomas E Dowling

**Affiliations:** School of Life Sciences, Arizona State University, 85287-4601 Tempe, AZ USA; Museu Nacional de História Natural e da Ciência, Centre for Ecology, Evolution and Environmental Changes (Ce3C), Universidade de Lisboa, Rua da Escola Politécnica 58, 1250-102 Lisbon, Portugal; Current address: Zoological Institute, University of Basel, 4051 Basel, Switzerland; Departamento de Biodiversidad y Biología Evolutiva, Museo Nacional de Ciencias Naturales, CSIC, c/José Gutiérrez Abascal 2, 28006 Madrid, Spain; Current address: Department of Biological Sciences, Wayne State University, 5047 Gullen Mall, 48202 Detroit, MI USA

**Keywords:** Speciation-with-gene-flow, Sympatry, Secondary contact, Introgression, Hybrid ecotype, *Barbus*, *Luciobarbus*

## Abstract

**Background:**

The evolution of species boundaries and the relative impact of selection and gene flow on genomic divergence are best studied in populations and species pairs exhibiting various levels of divergence along the speciation continuum. We studied species boundaries in Iberian barbels, *Barbus* and *Luciobarbus*, a system of populations and species spanning a wide degree of genetic relatedness, as well as geographic distribution and range overlap. We jointly analyze multiple types of molecular markers and morphological traits to gain a comprehensive perspective on the nature of species boundaries in these cyprinid fishes.

**Results:**

Intraspecific molecular and morphological differentiation is visible among many populations. Genomes of all sympatric species studied are porous to gene flow, even if they are not sister species. Compared to their allopatric counterparts, sympatric representatives of different species share alleles and show an increase in all measures of nucleotide polymorphism (S, *H*_d_, *K*, π and *θ*). High molecular diversity is particularly striking in *L. steindachneri* from the Tejo and Guadiana rivers, which co-varies with other sympatric species. Interestingly, different nuclear markers introgress across species boundaries at various levels, with distinct impacts on population trees. As such, some loci exhibit limited introgression and population trees resemble the presumed species tree, while alleles at other loci introgress more freely and population trees reflect geographic affinities and interspecific gene flow. Additionally, extent of introgression decreases with increasing genetic divergence in hybridizing species pairs.

**Conclusions:**

We show that reproductive isolation in Iberian *Barbus* and *Luciobarbus* is not complete and species boundaries are semi-permeable to (some) gene flow, as different species (including non-sister) are exchanging genes in areas of sympatry. Our results support a speciation-with-gene-flow scenario with heterogeneous barriers to gene flow across the genome, strengthening with genetic divergence. This is consistent with observations coming from other systems and supports the notion that speciation is not instantaneous but a gradual process, during which different species are still able to exchange some genes, while selection prevents gene flow at other loci. We also provide evidence for a hybrid origin of a barbel ecotype, *L. steindachneri*, suggesting that ecology plays a key role in species coexistence and hybridization in Iberian barbels. This ecotype with intermediate, yet variable, molecular, morphological, trophic and ecological characteristics is the local product of introgressive hybridization of *L. comizo* with up to three different species (with *L. bocagei* in the Tejo, with *L. microcephalus* and *L. sclateri* in the Guadiana). In spite of the homogenizing effects of ongoing gene flow, species can still be discriminated using a combination of morphological and molecular markers. Iberian barbels are thus an ideal system for the study of species boundaries, since they span a wide range of genetic divergences, with diverse ecologies and degrees of sympatry.

**Electronic supplementary material:**

The online version of this article (doi:10.1186/s12862-015-0392-3) contains supplementary material, which is available to authorized users.

## Background

The study of species boundaries provides invaluable information on the evolution of reproductive barriers and the impacts of gene flow on species divergence. This mechanistic approach is aimed at understanding how gene pools become subdivided and further gene flow is restricted or prevented. Gene exchange among taxa is likely to continue [1] and examples of divergence-with-gene-flow speciation [2] are now common in the literature. Speciation-with-gene-flow can range from divergence initiated in sympatry, to the evolution of additional isolating barriers after the establishment of secondary contact [3].

As taxa diverge, shared traits can reflect recent common ancestry and incomplete sorting of characters, reestablished gene exchange, or a combination of both. The two processes are difficult to separate, but the patterns they generate can potentially be discriminated using multiple independent markers and geographical information [4]. While traits sort and correlations among independent characters are generated as taxa diverge, gene flow breaks down those correlations. If introgressive hybridization is important, pre-existing correlations may be broken down by gene flow in sympatry but still persist in allopatry. Therefore, the signature of past isolation and divergence will be evident through the association among traits and geography. For instance, if species constitute monophyletic groups and only individuals in sympatry share alleles with other taxa, introgression rather than ancestral polymorphism is the likely cause of the pattern of allele sharing. Furthermore, allele sharing in areas of sympatry due to introgression is also expected to increase overall levels of molecular diversity, such as number of alleles and haplotype diversity, a pattern that is not likely to be generated by incomplete lineage sorting. Importantly, upon reestablishing some degree of gene flow the resulting patterns of variation will vary on a locus-by-locus basis, depending on impacts of selection and drift and on the architecture of the trait (*e.g.* [5–11]).

**Fig. 1:**
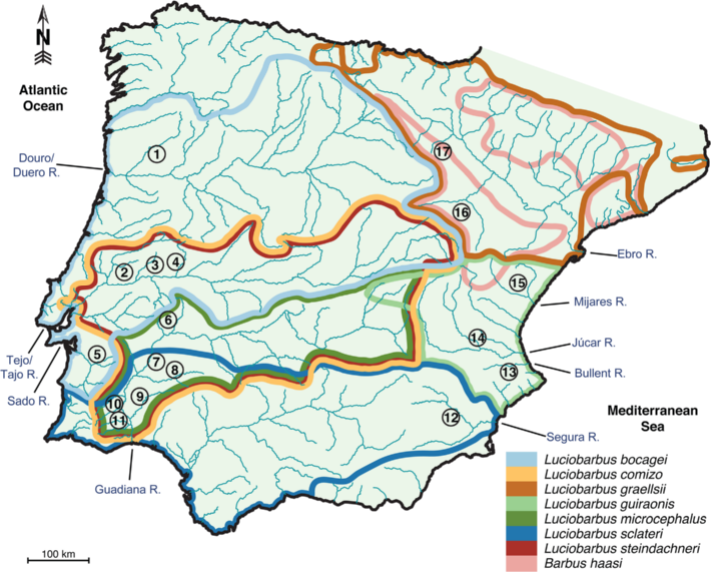
Map of the Iberian Peninsula showing major river drainages and distribution of barbel species. Color lines delimit the distribution ranges of each species. Collection sites: 1 – Tâmega; 2 – Zêzere; 3 – Ocreza; 4 – Erges; 5 – Xarrama; 6 – Caia; 7 – Guadiana; 8 – Ardila; 9 – Chança; 10 – Vascão; 11 – Foupana; 12 – Segura; 13 – Bullent; 14 – Cabriel; 15 – Mijares; 16 – Mesa; 17 – Alhama (see also Table 1)

The relative impacts of homogenizing gene flow and disruptive selection on species boundaries (*i.e.* genomic divergence) is most easily studied in recently speciated groups that show some degree of geographic overlap, such as finches, butterflies and lizards (*e.g.* [12–17]). Among taxa that present these ideal characteristics are the speciose sister genera *Barbus* and *Luciobarbus*. This group of pseudotetraploid fishes started diversifying throughout fresh waters of the circum-Mediterranean region more than 20 ma (million years ago) [18–20]. In the Iberian Peninsula, *Barbus* is represented by two species, *B. meridionalis* and the endemic *B. haasi*, while *Luciobarbus* contains seven endemic species (*L. bocagei*, *L. comizo*, *L. graellsii*, *L. guiraonis*, *L. microcephalus*, *L. sclateri*, and *L. steindachneri*; [21–26]), which started diversifying around 8 ma [18–20]. Different species are usually confined to specific ichthyogeographic provinces, inhabiting from one up to several river basins, and their ranges overlap to different extents (Fig. 1). This mostly allopatric distribution pattern indicates that speciation in Iberian barbels follows the evolution of river basins [23, 24, 26–28]. Secondary contacts in riverine species can be facilitated by several geomorphological processes affecting drainage patterns, such as river capture, marine regression and divide overtopping [29]. Allopatric diversification and range expansion with secondary contacts has been explicitly demonstrated for one polytypic species [30].

In diverse groups with varying degrees of range overlap such as barbels, species identification can be difficult. For instance, taxonomic status of *L. steindachneri* has been problematic due to its morphological variability and similarity to *L. comizo*, and has been considered a junior synonym of the latter by some authors (after [31]). Nevertheless, from an ecological standpoint it has been shown that *L. steindachneri* prefers habitats further upstream from those occupied by *L. comizo* and *L. microcephalus*, but lower than those inhabited by *L. sclateri* in the Guadiana River Basin [32–34]. *Luciobarbus steindachneri* also exhibits different trophic adaptations (*e.g.* intermediate mouth protrusion) and some degree of food partitioning relative to *L. bocagei* and *L. comizo* in the Tejo/Tajo River Basin [35–37] and *L. microcephalus* in the Guadiana River Basin [38]. Together, these data suggest that ecology plays a major role in species coexistence and has led some authors to instead regard *L. steindachneri* as an ecotype of *L. comizo* (*e.g.* [34]).

Despite coexistence, hybridization of Iberian barbels in areas of species overlap has been hypothesized based on specimens with intermediate phenotypes [21, 39–41] and mtDNA polyphyly [42], but inference of hybridization in these studies suffers from species identification problems [26] and circularity. In fact, hybridization in Iberian barbels has not been unequivocally demonstrated except in a few cases in which nuclear markers were examined [30, 43]. Unfortunately, most studies used a single type of character, precluding the examination of patterns of covariation among them. Furthermore, pseudotetraploidy of barbels limited these studies to the use of low-resolution nuclear markers, which in principle restricts their use to study more divergent species pairs. However, this limitation has been overcome with the development of paralog-specific primers for the amplification of nuclear loci in barbels [44].

Given that Iberian barbels have various degrees of divergence and geographic overlap, these species provide an excellent natural system to understand how reproductive barriers build up. We examine the morphological and genetic distinctiveness of all Iberian barbels, whether reproductive isolation is complete, and how patterns of gene flow vary among loci and species. To this end we use multiple classes of characters (external morphological traits, mitochondrial and nuclear DNA sequence data) and their patterns of covariation on the entire constellation of endemic *Barbus* (one species) and *Luciobarbus* (seven species), sampled from sympatric and allopatric populations (Table 1).

**Table 1:** Sample sizes of *Barbus* and *Luciobarbus* analyzed across different traits

Species	Basin	River	Region	Morphology	mtDNA	nuDNA
*L. bocagei*	Douro	Tâmega	Iberia	10	10	10
	Tejo	Erges, Zêzere	Iberia	12	12	12
	Sado	Xarrama	Iberia	6	10	10
*L. comizo*	Tejo	Erges, Ocreza	Iberia	7	7	7
	Guadiana	Guadiana, Ardila, Vascão	Iberia	8	8	8
*L. graellsii*	Ebro	Mesa	Iberia	10	10	10
*L. guiraonis*	Júcar	Cabriel	Iberia	10	10	10
	Mijares	Mijares	Iberia	6	8	8
	Bullent	Bullent	Iberia	8	0	0
*L. microcephalus*	Guadiana	Guadiana, Ardila, Chança	Iberia	14	14	14
*L. sclateri*	Guadiana	Chança, Foupana,	Iberia	11	11	11
	Segura	Segura	Iberia	10	10	10
*L. steindachneri*	Tejo	Erges, Ocreza	Iberia	14	14	14
	Guadiana	Guadiana, Ardila, Caia, Chança, Foupana	Iberia	26	26	26
*B. haasi*	Ebro	Alhama	Iberia	8	1	1
*B. barbus*	Vistula	Propad	Slovakia	0	3	3
	–	–	France	0	2	2
*B. carpathicus*	Vistula	Propad	Slovakia	0	3	3
*B. prespensis*	Lake Prespa	Agios Germanos	Greece	0	3	3
			*Total*	160	162	162

mtDNA: mitochondrial DNA; nuDNA: nuclear DNA

## Results

### Multivariate analysis of morphological data

**Fig. 2:**
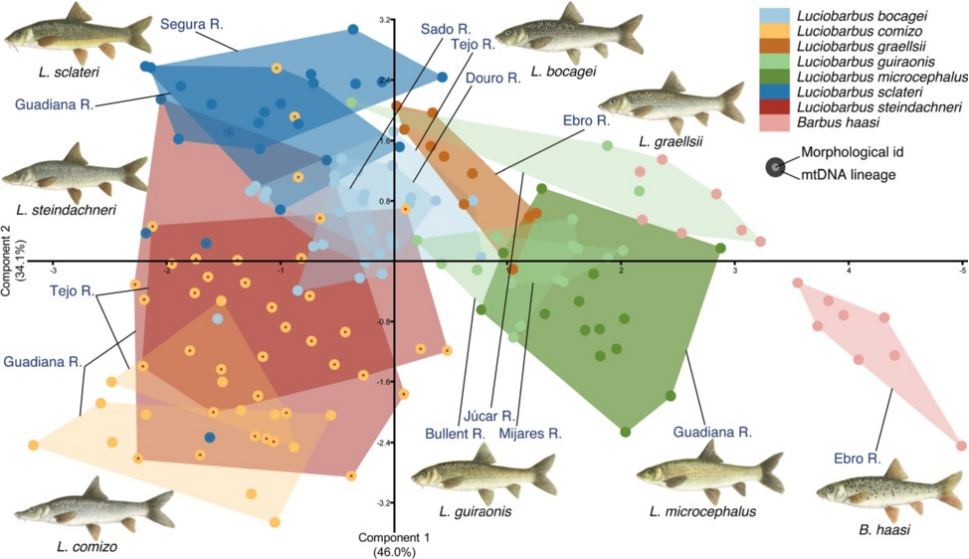
Scatterplot of PC1 and PC2 of meristic traits of all endemic Iberian *Barbus* and *Luciobarbus* species. Center of symbol represents morphological identification of specimens following Almaça [21], perimeter of each symbol represents mtDNA lineage defined in Fig. 3. Polygon delimits samples from each population/species

Multivariate analysis of meristic traits is effective in separating the Iberian endemic species of *Barbus* and *Luciobarbus*. The first two principal components explain 80.1 % of the observed variation, with all variables contributing to both axes, although traits that contribute the most to one axis contribute the least to the other (Table 2). Plotting the two components against each other provides clear visual separation of most of the recognized species (Fig. 2). *Barbus haasi* is the most morphologically distinct Iberian barbel, with all *Luciobarbus* analyzed being more similar to each other in meristic morphospace. The first component separates *L. comizo*, *L. bocagei* and *L. sclateri* from *L. microcephalus*, *L. guiraonis* and *L. graellsii*, and these groups from *B. haasi*, while the second component separates species within the first two groups of *Luciobarbus*. Therefore, species living in sympatry can be correctly discriminated using a few morphological traits, with the notable exception of *L. steindachneri*. Specimens of *L. steindachneri* show substantial variation in meristic traits, some individuals overlapping with either *L. comizo* or *L. bocagei* in the Tejo River and with *L. comizo* or *L. sclateri* in the Guadiana River. As a whole, *L. steindachneri* occupies an intermediate position in the morphospace relative to the other Iberian *Luciobarbus*.

**Table 2:** Principal components of meristic traits of all Iberian *Barbus* and *Luciobarbus* samples

Trait	PC1	PC2
LL	0.268	−0.544
TRA	0.332	−0.484
TRB	0.408	−0.375
IOC	−0.447	−0.370
SOC	−0.463	−0.311
POMC	−0.486	−0.313
Eigenvalue	2.76	2.05
Variance	46.0 %	34.1 %

Loadings of each original variable in components 1 and 2, eigenvalues and percentage of variance explained. LL: number of scales along the lateral line; TRA: number of scales in transverse rows above the LL; TRB: number of scales in transverse rows below the LL; SOC: supraorbital canal pores, IOC: infraorbital canal pores; POMC: preopercular-mandibular canal pores

Intraspecific differences are also observable, including between populations of *L. comizo* from the Tejo and Guadiana rivers, between populations of *L. guiraonis* from the Bullent and Mijares rivers relative to that from the Júcar River, and between populations of *L. sclateri* from the Guadiana and Segura rivers (albeit to a lesser degree). Populations of *L. guiraonis* from the Bullent and Mijares rivers overlap with each other and with *L. microcephalus* for these specific meristic traits (numbers of scales and cephalic pores); however, these taxa are readily discriminated by other traits, such as position of the mouth, shape of the dorsal fin and robustness of the last simple dorsal ray (Fig. 2 and [22]).

### Mitochondrial phylogeny

**Fig. 3:**
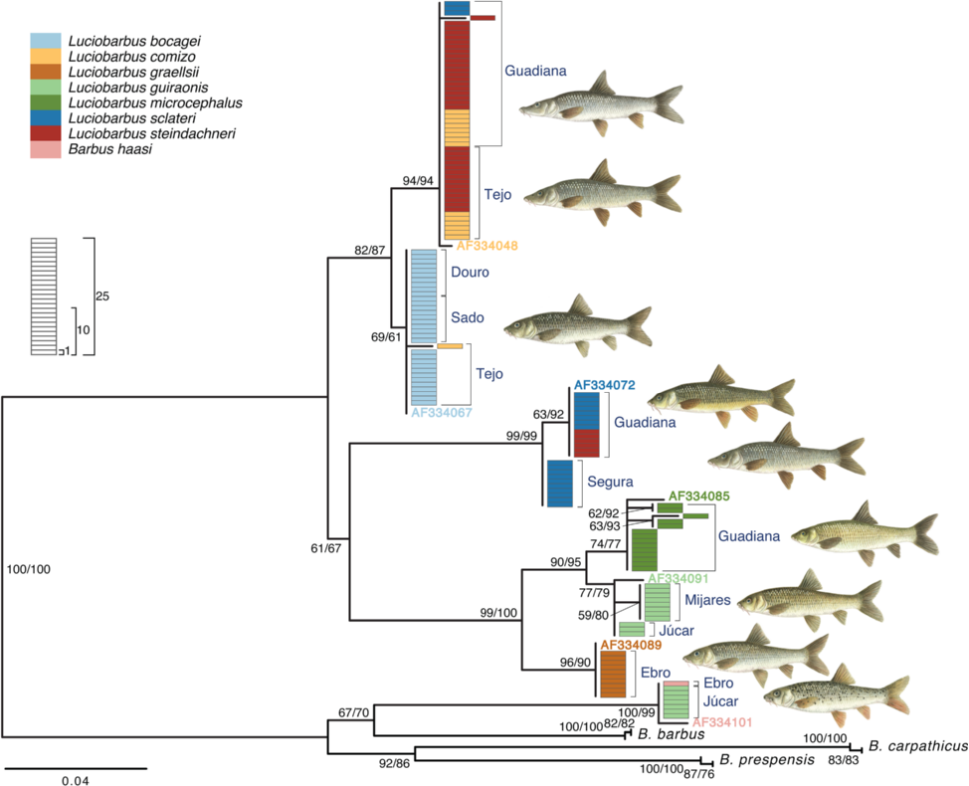
Maximum likelihood phylogeny of mitochondrial cyt *b* haplotypes. Bootstrap and Approximate Likelihood Ratio Test support values (left/right, respectively) are given next to relevant nodes. Each rectangle represents one sequence. Colors represent morphological identification of specimens following Almaça [21]

Analysis of 151 individuals representing the eight endemic barbel species identified 14 different cyt *b* haplotypes (Table 3), as found in previous studies [26, 45]. Two haplotypes are typical of *L. comizo*, two of *L. bocagei*, two of *L. sclateri*, two of *L. guiraonis*, four of *L. microcephalus*, one of *L. graellsii* and one of *B. haasi*. The Iberian *Luciobarbus* haplotypes form three main lineages, which are thought to reflect the true species tree. One lineage is composed of haplotypes found in the sister *L. comizo* and *L. bocagei*, which share a common ancestor with a second lineage, composed of the polytypic *L. sclateri*. The third lineage is composed of *L. graellsii* and the sister *L. microcephalus* and *L. guiraonis* (Fig. 3).

**Table 3:** Distribution of cyt *b* haplotypes across species and populations sampled

Species	Population	*A*	*B*	*C*	*D*	*F*	*H*	*J*	*K*	*M*	*N*	*S*	*T*	*X*	*Z*
*L. bocagei*	Douro		10												
*L. bocagei*	Tejo		12												
*L. bocagei*	Sado		10												
*L. comizo*	Tejo	1		6											
*L. comizo*	Guadiana			8											
*L. steindachneri*	Tejo			14											
*L. steindachneri*	Guadiana			19	1		6								
*L. microcephalus*	Guadiana							2	2	9	1				
*L. sclateri*	Guadiana			3			8								
*L. sclateri*	Segura					10									
*L. guiraonis*	Júcar											3			7
*L. guiraonis*	Mijares												8		
*L. graellsii*	Ebro													10	
*B. haasi*	Ebro														1

Mitochondrial haplotype codes follow [45]

Cytochrome *b* haplotypes are readily associated with a morphological species, again with the exception of individuals of *L. steindachneri* analyzed, which have mtDNA haplotypes typical of *L. comizo* or *L. sclateri*. In addition, a small number of specimens of several species possess mtDNA typical of other sympatric taxa, but never of allopatric taxa. This pattern is especially pronounced in *L. guiraonis* from the Júcar River where most specimens examined exhibit *B. haasi* mtDNA (Fig. 2).

### Nuclear gene phylogenies

**Fig. 4:**
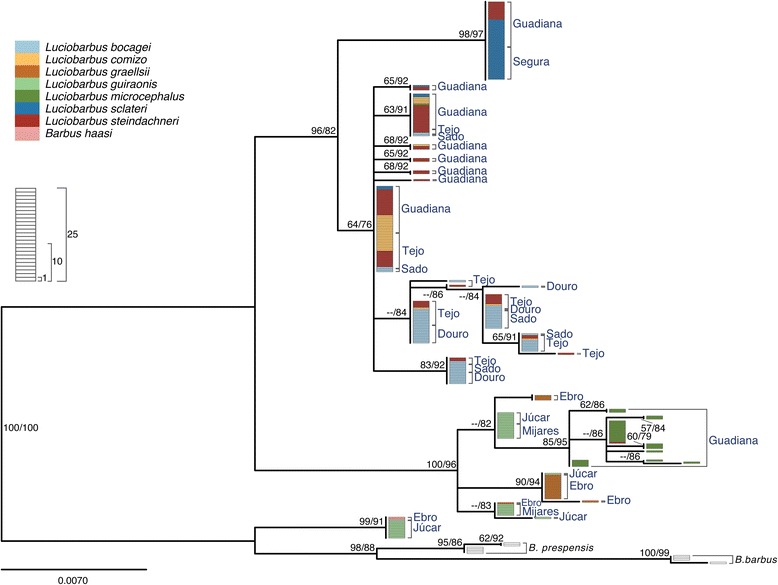
Maximum likelihood phylogeny of *S7-1* alleles. Bootstrap and Approximate Likelihood Ratio Test support values (left/right, respectively) are given next to relevant nodes. Each rectangle represents one allele. Colors represent morphological identification of specimens following Almaça [21]

**Fig. 5:**
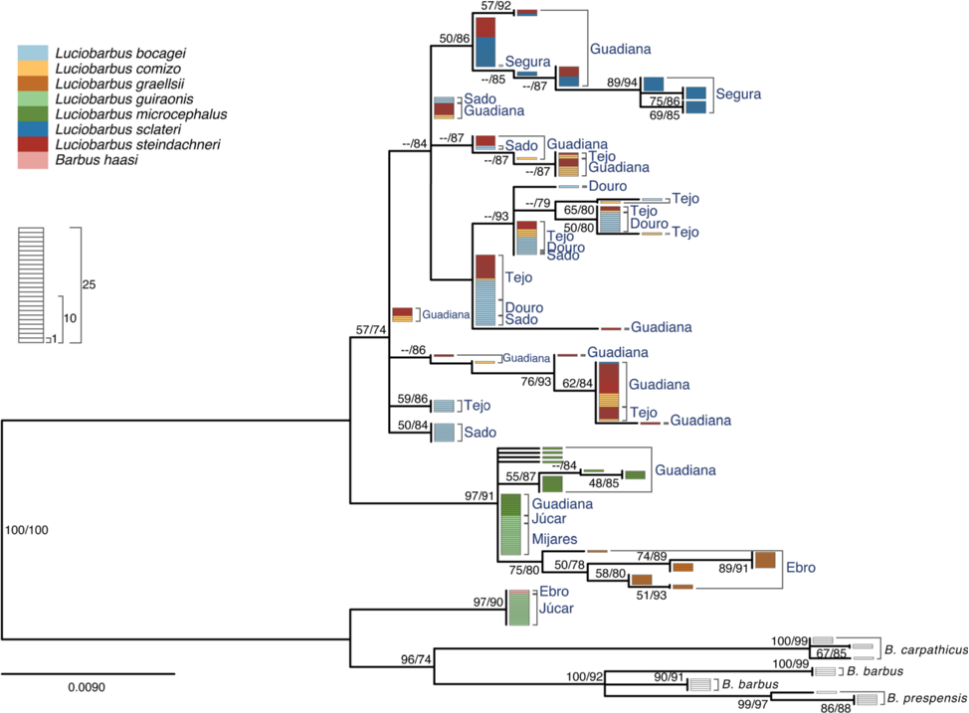
Maximum likelihood phylogeny of *S7-2* alleles. Bootstrap and Approximate Likelihood Ratio Test support values (left/right, respectively) are given next to relevant nodes. Each rectangle represents one allele. Colors represent morphological identification of specimens following Almaça [21]

**Fig. 6:**
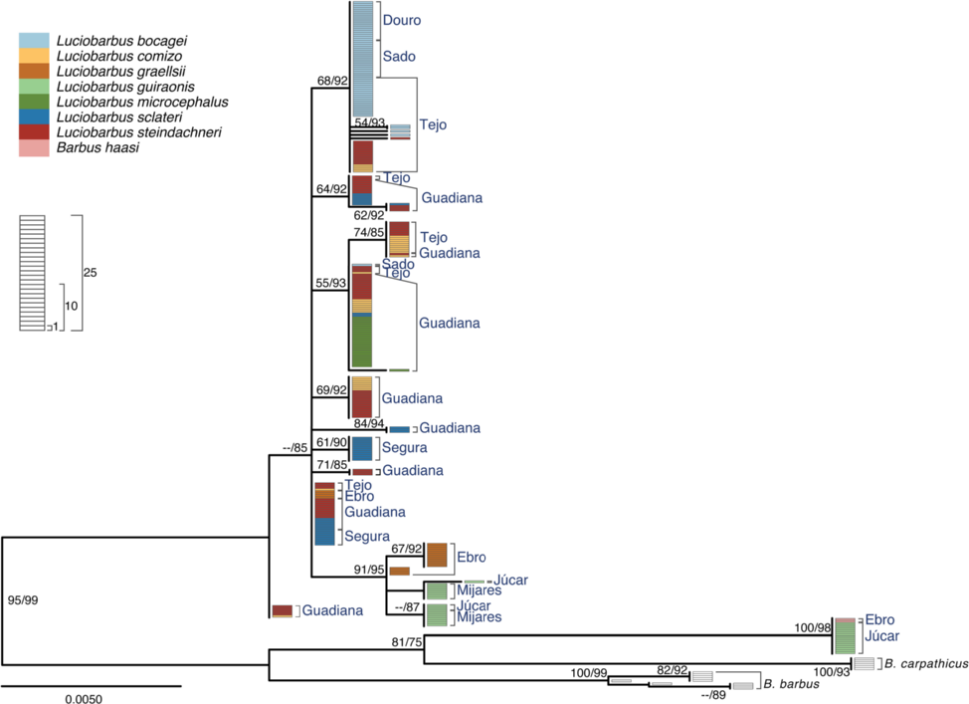
Maximum likelihood phylogeny of *Gh-1* alleles. Bootstrap and Approximate Likelihood Ratio Test support values (left/right, respectively) are given next to relevant nodes. Each rectangle represents one allele. Colors represent morphological identification of specimens following Almaça [21]

**Fig. 7:**
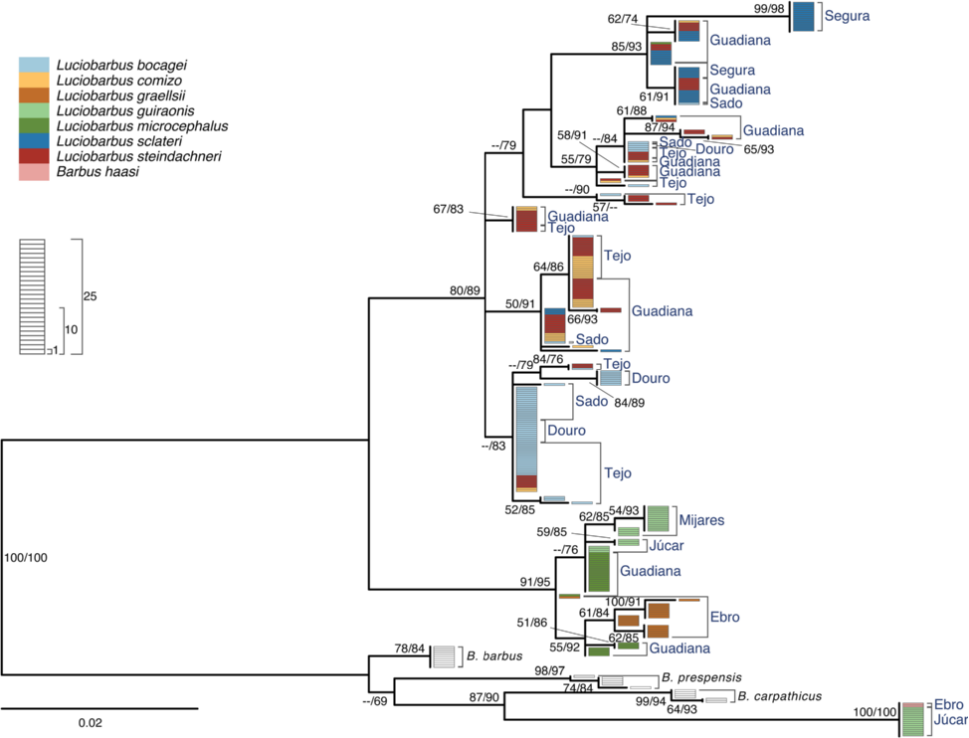
Maximum likelihood phylogeny of *Gh-2* alleles. Bootstrap and Approximate Likelihood Ratio Test support values (left/right, respectively) are given next to relevant nodes. Each rectangle represents one allele. Colors represent morphological identification of specimens following Almaça [21]

Sequence analysis of four nuclear loci yielded 3,792 aligned sites (*S7-1*, 813 bp; *S7-2,* 827 bp; *Gh-1,* 1018 bp; *Gh-2,* 1134 bp). The Iberian specimens analyzed exhibited 35**,** 54**,** 41 and 41 alleles for *S7-1*, *S7-2*, *Gh-1* and *Gh-2*, respectively. In general, gene trees are similar to each other (Figs. 4, 5, 6, 7) and are in agreement with relationships previously suggested by mtDNA, allozymes and morphology (*e.g.* [22–27, 30, 46, 47]). In particular, two main monophyletic lineages corresponding to the two genera, *Barbus* and *Luciobarbus*, are recovered. The former lineage is comprised of the Iberian endemic *B. haasi* and central and eastern European taxa included for phylogenetic context (*B. barbus*, *B. carpathicus* and *B. prespensis*). For *S7-1*, *S7-2* and *Gh-2*, the Iberian *Luciobarbus* lineage comprises two monophyletic groups, one composed of *L. graellsii*, *L. guiraonis* and *L. microcephalus*, and another composed of *L. bocagei*, *L. comizo*, *L. sclateri* and *L. steindachneri. Gh-1* is the least resolved nuclear marker at the species level, revealing a monophyletic group composed of *L. graellsii* and *L. guiraonis*, while *L. microcephalus* is recovered in a weakly supported group together with other sympatric species. The Iberian *Luciobarbus* lineages are further diagnosed by particular insertion-deletion variants at *S7-1*, *S7-2* and *Gh-2*.

The relationships within the two Iberian *Luciobarbus* lineages are less resolved, with most species para- or polyphyletic for some or all nuclear loci. As in the case of mtDNA, several specimens possess alleles typical of other sympatric species (Tejo and Guadiana rivers), or from allopatric taxa found in adjacent basins (Sado River). The most obvious case is represented by the two divergent lineages present in *L. guiraonis* from Júcar, one typical of *B. haasi* and the remaining also found in other populations of *L. guiraonis*. Notably, *L. steindachneri* is not distinct from the other sympatric species as most of its alleles are also found in *L. comizo* and *L. bocagei* from the Tejo River, and in *L. comizo*, *L. microcephalus* and *L. sclateri* from the Guadiana River.

### Population-level nuclear variation and differentiation

Levels of nuclear polymorphism vary substantially among populations, from five segregating sites (S) observed in *L. guiraonis* from Mijares, to 91 segregating sites observed in *L. guiraonis* from the Júcar River (Table 4). Nucleotide diversity (π) also differs by an order of magnitude among these populations, but most species/populations show intermediate levels of polymorphism (Table 4). Interestingly, allopatric populations of *Luciobarbus* exhibit lower levels of nucleotide polymorphism than sympatric populations (Mann–Whitney *U*-tests: *z* = 2.2736–2.7608, False Discovery Rate corrected *P =* 0.0072–0.0116, for all measures of polymorphism, S, *H*_d_, *K*, π and *θ*). In addition, both populations of *L. steindachneri* exhibit the highest numbers of segregating sites and levels of nucleotide diversity of sympatric species, in line with results from phylogenetic analyses. Additionally, discounting the effects of introgression, populations inhabiting smaller basins (*e.g.*, Mijares, Sado and Segura) show lower levels of nucleotide polymorphism than those inhabiting larger river basins as Guadiana or Tejo, consistent with differences in effective population size.

**Table 4:** Nuclear sequence polymorphism in Iberian barbel populations

Species	Population	No.	*S*	*H*_d_	*K*	*π*	*θ*
*L. bocagei*	Douro	20	15	0.92632	5.22632	0.00177	0.00127
*L. bocagei*	Tejo	24	26	0.98913	5.84420	0.00198	0.00219
*L. comizo*	Tejo	14	23	0.97802	6.95604	0.00236	0.00213
*L. steindachneri*	Tejo	28	29	0.99471	9.31481	0.00316	0.00232
*L. bocagei*	Sado	20	21	0.94737	4.51053	0.00153	0.00191
*L. comizo*	Guadiana	16	23	1.00000	7.65000	0.00259	0.00238
*L. steindachneri*	Guadiana	52	51	0.99698	11.13801	0.00377	0.00373
*L. microcephalus*	Guadiana	28	35	0.94444	4.67989	0.00159	0.00318
*L. sclateri*	Guadiana	22	29	0.99134	7.08225	0.00240	0.00256
*L. sclateri*	Segura	20	11	0.94211	4.19474	0.00142	0.00087
*L. guiraonis*	Júcar	20	91	0.70526	36.59474	0.01240	0.00888
*L. guiraonis*	Mijares	16	5	0.85000	2.51667	0.00085	0.00043
*L. graellsii*	Ebro	20	17	0.98421	5.18421	0.00176	0.00162
*B. haasi*	Ebro	2	0	0.00000	0.00000	0.00000	0.00000

No.: number of alleles; S: number of segregating sites; *H*_d_: haplotype diversity; *K*: average number of differences; π: nucleotide diversity; *θ*: Watterson estimator

To test how polymorphisms across loci are structured among populations we generated estimates of D_xy_, D_a_ and *F*_ST_ (Additional file 1) and clustered them using the neighbor-joining method (Additional file 2). Population trees built using estimates derived from mtDNA or all nuclear loci combined are generally consistent with phylogenetic results presented above. They identify two main groups, the genera *Barbus* and *Luciobarbus*. Within the latter, one subgroup is composed of *L. graellsii*, *L. guiraonis* and *L. microcephalus*, while the other contains *L. bocagei*, *L. comizo*, *L. sclateri* and *L. steindachneri*. Different populations of the same species are typically more similar to each other than to other species, with the exception of *L. steindachneri* and *L. guiraonis* (which reflect patterns of allele sharing detailed above). When nuclear loci are analyzed individually to investigate how nucleotide variation along different regions of the genome is shared across species and populations, different scenarios become clear, depending on the locus: *S7-1* and *Gh-2* follow the expected species phylogeny, while *S7-2* and *Gh-1* reflect geographical affinities. Thus, the latter genomic regions reflect geographic proximity and interspecific gene flow, while the former reflect phylogenetic affinities.

### Bayesian clustering of nuclear data

**Fig. 8:**
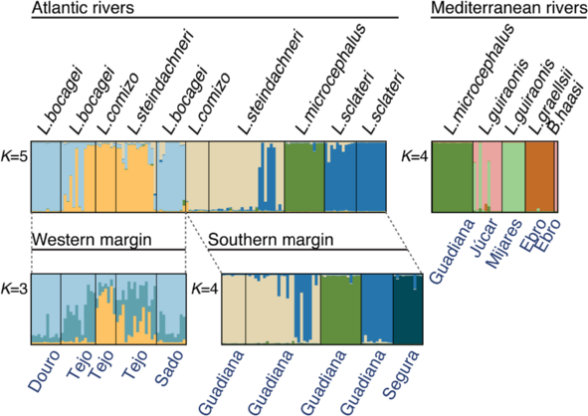
Bayesian analysis of population structure. Split-and-reanalyze strategy implemented in structure. The entire dataset was initially split into Atlantic- and Mediterranean-draining basins (top left and top right, respectively). The first dataset also includes a population of *L. sclateri* from a Mediterranean-draining basin (Segura R.) as an allopatric counterpart to *L. sclateri* from Guadiana R.. The Atlantic-draining dataset was subsequently split into west- and south-draining basins (bottom). Using a combination of changes in consecutive LnP(D) values and Evanno’s [48] Δ*K*, we determined the most likely number of clusters in each dataset. These analyses indicate that an overall *K* = 10 populations is the most biologically meaningful genetic structuring of the nuclear dataset. In Additional file 3 we present an alternative strategy to estimate *K*, using the run with highest likelihood value for each *K*. The results are very similar, with the detection of additional differentiation within *L. bocagei* (*K* = 11)

Due to the presence of large number of clusters (*K*) and high levels of differentiation among many populations and species, Structure sometimes converged to different solutions in independent replicates of each *K*, making determination of the best *K* challenging. Therefore, we consecutively split the complete dataset into smaller datasets, as recommended by the authors of the program as a strategy to deal with dataset multimodality. To determine the number of clusters we followed changes in LnP(D) values of consecutive *K* (*i.e.* when values plateau) and Evanno’s *et al.* Δ*K* [48]. We first split the dataset in two, based on geography and phylogenetic relationships. The first dataset is composed of all species from rivers draining to the Atlantic and allopatric populations of those species (in this case only *L. sclateri* from the Segura River). This analysis converged to *K* = 5 genetic clusters (Fig. 8, upper left), clearly separating populations inhabiting rivers draining to the western margin of the Iberian Peninsula (Douro, Tejo and Sado rivers) from rivers draining to the southern margin (Guadiana and Segura rivers). Further splitting the first dataset using this geographical discontinuity and patterns of gene exchange allows the identification of further intraspecific genetic structure (*K* = 3 and *K* = 4; Fig. 8, bottom). The second dataset is composed of species sympatric in rivers draining to the Mediterranean (Ebro, Júcar, Mijares and Bullent rivers) and closely related species (*i.e.*, *L. microcephalus* from the Guadiana River). This analysis converged to *K* = 4 genetic clusters (Fig. 8, upper right), which is concordant with species identification based on morphology. The only discordant samples are *L. guiraonis* from the Júcar River, which are placed in the same group as *B. haasi*.

Overall we identified 10 distinct population clusters, which allowed the genetic discrimination of almost all species included in the study, as well as allopatric populations of *L. comizo* and *L. sclateri*, and identification of within-population differentiation in *L. bocagei* from Tejo. Conversely, and in spite of clear power to diagnose even distinct intraspecific clusters, *L. steindachneri* does not constitute a genetically distinct unit. In turn, nuclear variation in *L. steindachneri* co-varies with sympatric species, in particular with *L. comizo* and *L. bocagei* in the Tejo River, and with *L. comizo* and *L. sclateri* (and *L. microcephalus* to a lesser extent) in the Guadiana River. This is another aspect noticeable from the Structure analyses, sympatric populations share more alleles across genetic clusters than allopatric ones, consistent with higher polymorphism levels in sympatry found above.

### Levels of gene flow across species boundaries of different ages

**Fig. 9:**
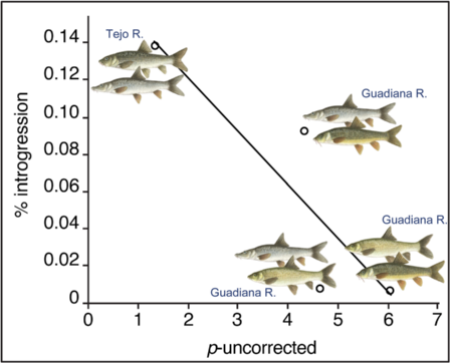
Relationship between levels of introgression and genetic divergence between hybridizing species pairs. A negative correlation between proportion of introgressed alleles and genetic divergence between species is observed, indicating a decrease in gene flow with increasing genetic differentiation. Species pairs in increasing order of genetic divergence based on mitochondrial DNA (from [26]): *L. bocagei*–*L. comizo*, *L. comizo*–*L. sclateri*, *L. microcephalus*–*L. sclateri*, *L. comizo*–*L. microcephalus*

We tested the hypothesis of gradual accumulation of genomic barriers to gene flow by examining the relation between the proportion of introgressed nuclear alleles (alleles shared only in areas of sympatry) between pairs of co-occurring species and their degree of divergence (see Methods section for details). We found that levels of gene flow across the species boundaries vary inversely with species divergence, *i.e.*, introgression is more reduced between more divergent species (*e.g.* between *L. microcephalus* and *L. sclateri*) than between more closely related ones (*e.g.* between *L. bocagei* and *L. comizo*; *r*^2^ = 0.773, Spearman’s Rank *ρ* = −1, *P* = 0.0416; Fig. 9).

## Discussion

**Fig. 10:**
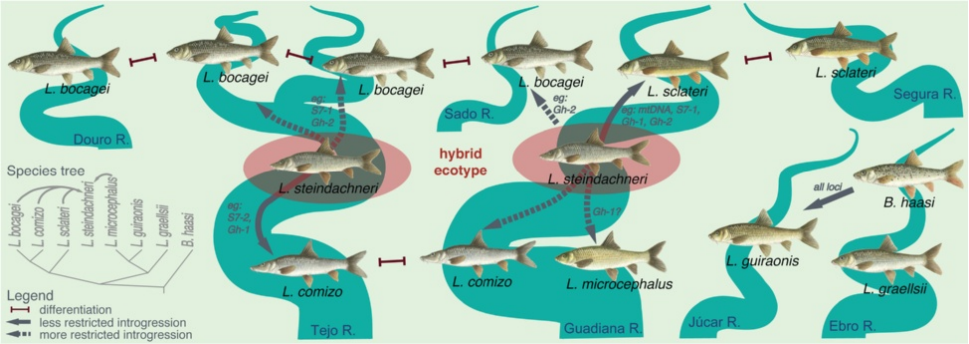
Semi-permeable species boundaries in Iberian barbels. Species boundaries in Iberian barbels are semi-permeable to gene flow in areas of sympatry after an initial period of allopatric differentiation. Genetic data support a speciation-with-gene-flow scenario with levels of interspecific gene flow inversely associated with divergence, as represented by solid or dashed arrows. Traits involved in ecological preference (*e.g.* habitat, diet) are likely contributing to reproductive isolation and species coexistence. When these breakdown an ecotype of hybrid origin (*L. steindachneri*) is formed, frequent in areas with intermediate habitat characteristics

It has been suggested that studying pairs of populations and species spanning the speciation continuum can contribute more comprehensively to our understanding of how selection affects genomic divergence [9]. Our study successfully combines the analyses of populations and species spanning a wide degree of divergence, geographic distribution and range overlap, with different molecular markers and morphological traits, which provides exceptional perspective on the nature of species boundaries. Such a holistic approach has proven very informative in other systems (*e.g.* [49–54]). We show that species boundaries in Iberian *Barbus* and *Luciobarbus* are semi-permeable, as different species (including non-sister taxa) are exchanging genes in areas of sympatry after an initial period of allopatric differentiation. Distribution of genetic variation across space, loci and species supports a speciation-with-gene-flow scenario with levels of interspecific gene flow inversely associated with divergence. We also provide evidence for a hybrid origin of a barbel ecotype, *L. steindachneri*, suggesting that ecology plays a key role in species coexistence and hybridization in Iberian barbels (Fig. 10).

### Speciation-with-gene-flow in Iberian barbels

Patterns of molecular variation suggest that genomic differentiation in barbels generally starts in allopatry. It has been suggested that *Luciobarbus* evolution follows river basin evolution as sister species usually inhabit one or a few adjacent river basins [23, 24, 26–28]. Gante *et al.* [30] showed that polytypic *L. sclateri* diversified in allopatry and expanded its distribution westwards in southern Iberia, leading to secondary contacts with *L. comizo* and *L. microcephalus* in the Lower Guadiana River less than 500 ka (thousand years ago). Geographical scenarios for the evolution of all other Iberian barbels are not equally detailed, but our data indicate that intraspecific differentiation in allopatry is the norm: in addition to discrimination at the species levels, geographic (allopatric) differentiation is also found where samples from multiple basins are available (*L. bocagei*, *L. comizo* and *L. sclateri*). It is therefore reasonable to assume that along the speciation continuum the same mechanisms act during differentiation in allopatry and are involved in all levels of differentiation, intra- and interspecific. We also observed within-basin differentiation in *L. bocagei*, which could be the result of its preference for low-order rivers as observed in rheophilic *Barbus* [20].

Contrasting patterns of molecular variation between areas of allopatry and sympatry is pivotal in excluding ancestral polymorphism as an explanation for the observed allele sharing and gene paraphyly between co-occurring species of Iberian *Barbus* and *Luciobarbus*. Individuals are generally readily assignable to a monophyletic group representative of each species, with only individuals in sympatry sharing alleles with other taxa. This leads to higher levels of nucleotide polymorphism in sympatric populations of different species relative to their allopatric counterparts, consistent with a role for gene flow in generating these spatial patterns of molecular variation. Geographical information has also proved important in discriminating between these processes in other young systems, such as sunflowers, fruit flies, *Heliconius* butterflies and cichlid fishes (*e.g.* [4, 55–57]). It is clear that reproductive isolation is not yet fully complete in Iberian barbels, including between non-sister species (*e.g. L. comizo* and *L. sclateri*), indicating that genomes remain porous and allow for gene exchange, even between genera that diverged more than 20 ma [18–20]. Incomplete reproductive isolation seems to be the norm in other diverse systems in which species can be found in sympatry, including other barbels [20, 58–67]. Multiple characteristics make freshwater fish generally prone to hybridize [68]. In barbels, natural characteristics associated to external fertilization include spawning migrations and group spawning during which multiple males arrive to breeding grounds and await incoming females. In addition, the likely asymmetrical behavioral isolating mechanisms and unequal abundance of the co-occurring species increase the likelihood of interspecific gene flow. Acting alone or in combination, incorrect species recognition or sneaking behavior by small males might explain why often mispairings involve females of the largest species [20, 69, 70]. Pseudotetraploidy and long generation times (*i.e.* slow evolutionary rates) might also buffer against possible effects of intrinsic genomic incompatibilities and slow down the evolution of reinforcement mechanisms, which would allow introgression between genera separated by more than 20 ma of evolution [18–20]. Anthropogenic sources of decreased habitat complexity and construction of obstacles preventing spawning migrations to correct breeding sites (such as reservoirs and dams) might also increase the chances of hybridization. Although it is likely that anthropogenic activities have influenced the extent of hybridization and directionality of introgression we currently observed, it is probable that some interspecific gene flow has occurred soon after species became sympatric due to changes in drainage geometry. Nevertheless and in spite of ongoing gene flow in areas of sympatry, Iberian barbels can be distinguished using a combination of morphological and molecular data, which suggests that reproductive barriers, albeit variable in strength, do exist for at least some parts of the genome.

How then do reproductive barriers become established? Theory predicts that loci involved in adaptation to different niches and speciation should show increased differentiation while neutral or nearly neutral loci not tightly linked to them should move more freely between populations and species [6, 71]. Therefore, genealogies derived from neutral or nearly neutral loci will be more susceptible to the impacts of interspecific gene flow than gene trees based on loci under divergent selection, which are prevented from introgressing and should better reflect species histories predating introgression after secondary contact. Additionally, frequency of new mutations is more likely to increase if they arise in regions of low introgression, potentially leading to a feedback loop of increased genomic differentiation [1]. Thus boundaries between older species pairs, which have accumulated more genetic differences, will likely exhibit greater genetic incompatibility and be less permeable to gene flow. We observe both scenarios in Iberian barbels: introgression across species boundaries varies among loci and gene flow seems to decay with increased divergence between species pairs, such that boundaries become less diffuse and more easily identifiable in more divergent, older species pairs. Therefore, reproductive isolation is not a property of the entire genome, nor does it arise instantaneously (*e.g.* [6]). Instead, these patterns are consistent with genetic differentiation (*i.e.* barriers to gene flow) starting in more or less localized genomic regions and then accumulating along the genome as species diverge [6, 9, 11, 58, 71–81]. Such ‘islands’ or ‘continents’ of the genome under stronger disruptive selection would exhibit reduced levels of introgression among species, relative to nearly neutral regions, retaining phylogenetic signal without noise from gene exchange among lineages. The relative impact of these two forces gradually changes with divergence until the influence of introgression is eventually ceased in all genomic regions. The precise mechanisms by which genomes become less porous and the signatures they leave behind are a major focus of speciation research [11, 79, 82–84]. Thus, all lines of evidence suggest that population differentiation and speciation in Iberian barbels are initiated in allopatry and accumulate as populations diverge. However, reproductive barriers are not (yet) sufficient to fully prevent gene flow where species meet in regions of secondary contact, as seen in other species of barbels [20, 69, 85]. In addition to levels of introgression differing between species pairs, the environment is also expected to play a role in hybridization outcomes in particular when ecology influences their degree of sympatry. Possible environmental effects on hybridization dynamics could be disentangled from genomic divergence effects by using replicated hybrid zones of the same species pairs.

### *Luciobarbus steindachneri* is a hybrid ecotype

The above considerations about species delimitation and mode of evolution fit all except for one nominal species, *L. steindachneri*. Its taxonomy has proved challenging since its description, as morphological and mtDNA similarity with *L. comizo* has already been identified [26, 31]. Here we provide evidence that explains these difficulties. Nuclear sequence data examined indicate that the genome of *L. steindachneri* shares its alleles across genetic clusters, mostly between *L. comizo* and all other sympatric species, both in the Tejo and Guadiana river basins. In addition, it has the highest numbers of segregating sites and levels of nucleotide diversity of sympatric species, which altogether support a scenario of admixture. It has the highest morphological variance of all Iberian barbels and is intermediate between several species, not only in meristic traits as seen in the present study and [21] but also in trophically relevant ones, such as mouth shape and position [35–37]. The latter are most likely under selection and have important fitness consequences [86, 87]. Differences in feeding apparatus likely explain observed food partitioning relative to *L. bocagei* and *L. comizo* in the Tejo River [35–37] and *L. microcephalus* in the Guadiana River [38], where it occupies an intermediate trophic position. Although *L. steindachneri* is always found in sympatry with other species, it prefers intermediate habitats and medium order rivers: it inhabits more frequently river stretches further upstream from those preferred by *L. comizo* and *L. microcephalus*, but downstream from typical *L. sclateri* habitat in the Guadiana River [32–34] and downstream from *L. bocagei* in the Tejo River [Gante pers. obs.]. Observed habitat preferences likely reflect selection on trophic morphology and trophic niche. These lines of evidence suggest that ecology plays a major role in the coexistence and hybridization among sympatric barbel species. *Luciobarbus steindachneri* is thus the local product of introgressive hybridization between *L. comizo* and *L. bocagei* in the Tejo, and *L. microcephalus* and *L. sclateri* in the Guadiana, for which we have no evidence to be a stabilized hybrid species. Therefore, following Morán-López *et al.*’s [34] terminology, we show that *L. steindachneri* is an ecotype of hybrid origin that is using a niche intermediate to those of its parents [88]. In spite of this general intermediacy, whether its ecology varies between the Tejo and Guadiana rivers, and with parental crosses involved has not been assessed but is likely to be the case. Overall these findings raise questions on how one of the most common barbels found in Iberian rivers should be dealt with from taxonomic and conservation perspectives, as it is of hybrid origin and not stabilized. Conversely, it becomes a great model in evolutionary ecology. Sampling of replicated hybrid zones would help elucidate how stable this hybrid is and other aspects, such as geographical differences in parental contribution and levels of hybridization.

## Conclusions

A fundamental question in speciation research is the evolution of barriers to gene flow and the long-term persistence of taxa in sympatry. We provide evidence for semi-permeable species boundaries in Iberian *Barbus* and *Luciobarbus* (summarized in Fig. 10). Different species are exchanging genes in areas of sympatry after an initial period of allopatric differentiation. Genomic barriers to gene flow are heterogeneous in strength and accumulate with increased divergence. Particularly puzzling is the case of intergeneric hybrids in the Júcar River, where only a hybrid swarm was found. Additional sampling outside this area would be necessary to tease apart possible causes for the apparent collapse or absence of isolating mechanisms. We also show that *L. steindachneri* is an ecotype of hybrid origin, suggesting that ecology plays a key role in species coexistence and hybridization. Since Iberian barbels span a wide range of genetic divergence, diverse ecologies, and degrees of sympatry, they provide an ideal opportunity for studying the evolution of reproductive isolation mechanisms. Given the complexities of this system (*i.e.*, ancient polyploidy) recent improvements in technology have made it possible to study systems that were traditionally less tractable due to technical challenges. In particular, newer deep sequencing technologies will allow further insight into the patterns of genomic divergence in the face of gene flow and mechanisms shaping reproductive barriers in barbels by sampling many more loci than the ones surveyed here. For instance, it would allow determining which genomic regions are responsible for reproductive isolation and when they arose relative to the establishment of secondary contacts.

## Methods

### Specimen collection and sample processing

Specimens of Iberian *Barbus* and *Luciobarbus* were collected by electrofishing throughout the species’ ranges (Fig. 1). All specimens were formalin fixed and ethanol preserved, and tissues ethanol preserved and deposited in the zoological collections ‘Museu Bocage’ (MB) of Museu Nacional de História Natural e da Ciência, Portugal, and in Museo Nacional de Ciencias Naturales (MNCN), Spain. Some museum samples were used only for morphological analyses, while others from central and eastern Europe were used only to provide context in phylogenetic analyses of molecular characters (Table 1).

We identified individuals to species based on Almaça’s [21] qualitative traits, such as dorsal fin shape, robustness of the last dorsal spine, head shape, eye and mouth position, barbel length and coloration of body and fins. Assignment of individuals to *L. comizo* and *L. steindachneri* can be difficult due to morphological similarity that has motivated their synonymy [31]; conservatively only specimens with clear phenotypes were assigned to *L. comizo*. Covariation of meristic morphological traits and molecular markers (see below) would reject the null hypothesis of ‘no species differentiation’ and confirm that qualitative traits are truly representative of species identity [89].

### Choice, scoring and analysis of morphological traits

For testing species discrimination using morphological traits, we chose additional independent characters based on their potential information content, scoring reliability and reproducibility, their consistent preservation in different conditions and ease of use. For these reasons, external meristic traits were preferred over morphometric traits, as the latter are further complicated by allometry, which allow us to include also juvenile specimens in the study. Even though there is substantial overlap in scale counts across different *Luciobarbus* species (*e.g.* [21, 31]), they vary widely and also exhibit different modal values. Three different scale counts were taken: number of scales along the lateral line (LL); number of scales in transverse rows above the LL, counted anteriorly-posteriorly from the LL to the sagittal line in front of the first dorsal-fin ray, not including the LL (TRA); number of scales in transverse rows below the LL, counted anteriorly-posteriorly from the LL to the insertion of the first pelvic-fin ray, not including the LL (TRB). Allowance was made for abnormal scale development, such as duplicated or fused scales, evaluated by comparison with scales from rows above and below the scales in question. Numbers of cephalic canal pores found to be useful in the diagnosis of *Barbus* species [90] were also examined, including those on the supraorbital canal (SOC), the infraorbital canal (IOC) and the preopercular-mandibular canal (POMC). Counts were taken preferentially on the left side under a dissecting scope using Cyanine Blue 5R temporary stain to enhance contrast of structures [91].

Covariation among morphological traits was assessed using principal components analysis (PCA), as implemented in PAST [92]. PCA explores co-linear variation of the original variables, reducing multidimensionality of the data into new orthogonal variables (principal components). The variance-covariance matrix of standardized meristic variables was used.

### Scoring and sequencing of mitochondrial and nuclear loci

The mitochondrial gene cytochrome *b* (cyt *b*) has proved to be useful in phylogenetic and phylogeographic studies of *Barbus* and *Luciobarbus* (*e.g.* [24, 26, 47, 93]). We screened for single-stranded conformational polymorphisms (SSCPs) of a 275 bp fragment following methods in Gante *et al.* [45]. Sequences of multiple (>10 %) representative SSCP bands from each gel were confirmed by Sanger sequencing on an ABI 3730 DNA Analyzer.

Several PCR primers for nuclear loci have become available for cyprinid fishes. Due to pseudotetraploidy of barbels, paralog-specific primers were used following Gante *et al.* (2011). Briefly, we used a hybrid annealing strategy, combining both universal exon-primed intron-crossing (EPIC) and paralog-specific intron-primed exon-crossing (IPEC) primers for amplification and sequencing of four nuclear loci: *S7-1*, *S7-2*, *Gh-1* and *Gh-2*. Sequences were obtained by Sanger sequencing on an ABI 3730 DNA Analyzer. Different methods were employed to resolve haplotypic phase of heterozygous individuals. Where individuals were heterozygous for insertions or deletions (indels), haplotypes were either manually phased using the method described by Flot *et al.* [94] or using the program Champuru [95]. Haplotypes with known phases were subsequently used to phase the single nucleotide polymorphism (SNP) heterozygotes with Phase [96]. Phase input files were generated using SeqPhase [97]. Consistency of the inferred haplotypes was assessed in five independent Phase runs, each of 100 iterations, burn-in of 100 and thinning interval of 1.

### Phylogenetic analyses of nucleotide sequences

The models of nucleotide sequence evolution for the different datasets were identified with jModeltest 2 [98, 99] using the corrected Akaike and Bayesian Information Criteria. Maximum likelihood allele phylogenies were built in PhyML 3.0 [100] using an HKY85 model of nucleotide evolution with four substitution rate categories, estimated transition/transversion ratios, with a proportion of invariable sites and gamma distribution parameter, and using empirical nucleotide frequencies. Previous published complete cyt *b* sequences of all species [26] were included in the analysis to check relationships of haplotypes found in the present study. Topology searches used NNI (nearest-neighbor interchange) and SPR (subtree pruning and regrafting) on a BioNJ starting tree. Node support was estimated using 1000 bootstrap replicates and approximate Bayes likelihood ratio tests.

### Levels of nucleotide polymorphism and population differentiation

Number of segregating sites (S), haplotype diversity (*H*_d_), average number of differences (*K*), nucleotide diversity (π) and Watterson estimator (*θ*), and pairwise population divergence measures (D_a_, D_xy_ and *F*_ST_) were calculated using the program DnaSP v5.10.01 [101]. Neighbor-joining (NJ) networks of pairwise population differentiation statistics were constructed in MEGA 5.05 [102] for each locus separately and all nuclear loci combined. Node support for each network was assessed by bootstrapping nucleotide sequence alignments (100 times) in Seqboot from the Phylip package [103] and re-calculating estimates.

### Bayesian clustering of nuclear DNA data

Covariation among nuclear loci was assessed using the Bayesian clustering program Structure v2.3.3 [104, 105]. Structure identifies clusters by assigning individuals to *K* populations in a way that minimizes deviations from Hardy–Weinberg equilibrium within clusters and maximizes linkage disequilibrium between them (*i.e.* species). Each unique allele was identified using the program Macclade v4.03 [106] and numerically coded (*i.e.* we coded each unique combination of SNPs at each locus and not each individual SNP). Due to the complexity (multimodality) of the complete nuclear dataset, Structure sometimes converged to different solutions in independent runs for the same *K*. Therefore, smaller, more tractable datasets consisting of combinations of sympatric and sister species and allopatric references were analyzed independently as recommended by Pritchard & Wen [107]. This approach allows for assessment of the importance of introgression and ancestral polymorphism on allele sharing. To assess reliability of solutions, 20 iterations were run for each *K*. Each iteration consisted of 500,000 MCMC (Markov Chain Monte Carlo) generations as burn-in, followed by 1,000,000 MCMC replicates to estimate the posterior sample distribution, using the admixture and correlated allele frequency models. LnP(D), the probability of the data given *K*, was tracked over the course of the burn-in and the run to ensure that these values had stabilized by the end of the burn-in period. Two different methods were used to determine the number of groups (*K*) identified by Structure runs of each dataset. The first method, suggested by Pritchard & Wen [107], identifies the most likely value of *K* by comparing changes in LnP(D) values of consecutive *K* (*i.e.* when values plateau). The second method, developed by Evanno *et al.* [48] finds the ad hoc quantity based on the second order rate of change of the likelihood function with respect to *K* (Δ*K*). Plots of these two metrics were obtained using Structure Harvester [108]. Consensus clustering across iterations for each for each K was generated using the greedy algorithm in clumpp [109] and visualized using the program distruct [110]. In addition, we present and discuss an alternative strategy to determine the best *K* using the complete dataset in the supplementary information (Additional file 3).

### Levels of introgression between pairs of hybridizing species

To test the hypothesis that reproductive barriers accumulate in the genome as species diverge, we calculated the proportion of introgressed alleles between pairs of hybridizing taxa. We restricted the analysis to pairs of species that we see from our data to co-occur and exchange genes in sympatry to avoid obscuring any patterns with zero-inflation from non-hybridizing species pairs (*e.g.* because they are never in sympatry). We also excluded the intergeneric hybridizing pair *B. haasi* and *L. guiraonis*, as ‘pure’ *B. haasi* has not yet been collected in the Júcar River. It is not presently clear if it inhabits some area of the basin yet to be sampled or if it went locally extinct. Therefore we analyzed the hybridizing pairs *L. bocagei*–*L. comizo*, *L. comizo*–*L. sclateri*, *L. microcephalus*–*L. sclateri* and *L. comizo*–*L. microcephalus*. Diagnostic alleles were identified from nuclear allele phylogenies: alleles shared only in sympatric populations of species A, but absent in their allopatric counterparts while common in sympatric or allopatric populations of species B were considered typical of the latter. Because *L. microcephalus* is fixed for *Gh-1* and inhabits only the Guadiana River we do not have allopatric samples to helps us distinguish whether it shares *Gh-1* alleles through introgression and complete replacement, or by retained ancestral polymorphism, we conservatively excluded this locus from comparisons involving this species. We used pairwise uncorrected mean divergence between taxa (*p*-uncorrected) derived from mtDNA as a surrogate for levels of species divergence (from [26]). Therefore, this estimate of divergence between taxa is independent from nuclear introgression levels determined in the present study and should better reflect species divergence in the absence of interspecific gene flow.

### Ethics statement

Direcção Geral dos Recursos Florestais (DGRF) provided the necessary collection permits. Fish were euthanized with an overdose of MS-222 (3-aminobenzoic acidethyl ester methanesulfonate) prior to handling.

## Availability of supporting data

The datasets supporting the results of this article are deposited in the Dryad Digital Repository [http://dx.doi.org/10.5061/dryad.m1572] and include five sequence alignment files and one morphological (meristic) traits file [111].

## Additional files

Additional file 1:
**Divergence measures across population pairs of Iberian barbels.**
Additional file 2:
**Population polymorphisms networks across loci.**
Additional file 3:
**STRUCTURE analysis using different numbers of samples.**

## References

[1] Gavrilets S: Fitness Landscapes and the Origin of Species. Princeton, New Jersey: Princeton University Press; 2004.

[2] Rice WR, Hostert EE (1993). Laboratory experiments on speciation: what have we learned in 40 years?. Evolution (N Y).

[3] Smadja CM, Butlin RK (2011). A framework for comparing processes of speciation in the presence of gene flow. Mol Ecol.

[4] Noor MAF, Bennett SM (2009). Islands of speciation or mirages in the desert? Examining the role of restricted recombination in maintaining species. Heredity (Edinb).

[5] Rieseberg LH, Whitton J, Gardner K (1999). Hybrid zones and the genetic architecture of a barrier to gene flow between two sunflower species. Genetics.

[6] Wu C-I (2001). The genic view of the process of speciation. J Evol Biol.

[7] Machado CA, Kliman RM, Markert JA, Hey J (2002). Inferring the history of speciation from multilocus DNA sequence data: the case of *Drosophila pseudoobscura* and close relatives. Mol Biol Evol.

[8] Bull V, Beltrán M, Jiggins CD, McMillan WO, Bermingham E, Mallet J (2006). Polyphyly and gene flow between non-sibling *Heliconius* species. BMC Biol.

[9] Nosil P, Funk DJ, Ortiz-Barrientos D (2009). Divergent selection and heterogeneous genomic divergence. Mol Ecol.

[10] Carneiro M, Ferrand N, Nachman MW (2009). Recombination and speciation: loci near centromeres are more differentiated than loci near telomeres between subspecies of the European rabbit (*Oryctolagus cuniculus*). Genetics.

[11] Nosil P, Feder JL (2012). Genomic divergence during speciation: causes and consequences. Philos Trans R Soc Lond B Biol Sci.

[12] Sato A, O’hUigin C, Figueroa F, Grant PR, Grant BR, Tichy H (1999). Phylogeny of Darwin’s finches as revealed by mtDNA sequences. Proc Natl Acad Sci.

[13] Beltrán M, Jiggins CD, Bull V, Linares M, Mallet J, McMillan WO (2002). Phylogenetic discordance at the species boundary: comparative gene genealogies among rapidly radiating *Heliconius* butterflies. Mol Biol Evol.

[14] Shaw KL (2002). Conflict between nuclear and mitochondrial DNA phylogenies of a recent species radiation: what mtDNA reveals and conceals about modes of speciation in Hawaiian crickets. Proc Natl Acad Sci U S A.

[15] Heckman KL, Mariani CL, Rasoloarison R, Yoder AD (2007). Multiple nuclear loci reveal patterns of incomplete lineage sorting and complex species history within western mouse lemurs (*Microcebus*). Mol Phylogenet Evol.

[16] Mullen SP, Dopman EB, Harrison RG (2008). Hybrid zone origins, species boundaries, and the evolution of wing-pattern diversity in a polytypic species complex of North American admiral butterflies (Nymphalidae: *Limenitis*). Evolution.

[17] Pinho C, Harris DJ, Ferrand N (2008). Non-equilibrium estimates of gene flow inferred from nuclear genealogies suggest that Iberian and North African wall lizards (*Podarcis* spp.) are an assemblage of incipient species. BMC Evol Biol.

[18] Gante HF: Diversification of circum-Mediterranean barbels. In Chang Divers Chang Environ. Edited by Grillo O, Venora G. Rijeka, Croatia: InTech; 2011:283–298.

[19] Levin BA, Freyhof J, Lajbner Z, Perea S, Abdoli A, Gaffaroğlu M (2012). Phylogenetic relationships of the algae scraping cyprinid genus *Capoeta* (Teleostei: Cyprinidae). Mol Phylogenet Evol.

[20] Buonerba L, Zaccara S, Delmastro GB, Lorenzoni M, Salzburger W, Gante HF: Intrinsic and extrinsic factors act at different spatial and temporal scales to shape population structure, distribution and speciation in Italian Barbus (Osteichthyes: Cyprinidae). Mol Phylogenet Evol. 2015;89:115–129. 10.1016/j.ympev.2015.03.024 25882833

[21] Almaça C (1967). Estudo das populações portuguesas do gén. *Barbus* Cuvier, 1817 (Pisces, Cyprinidae). Rev da Fac Ciências, Lisboa, 2a série.

[22] Doadrio I (1990). Phylogenetic relationships and classification of western palaearctic species of the genus Barbus (Osteichthyes, Cyprinidae). Aquat Living Resour.

[23] Zardoya R, Doadrio I (1998). Phylogenetic relationships of Iberian cyprinids: systematic and biogeographical implications. Proc Biol Sci.

[24] Zardoya R, Doadrio I (1999). Molecular evidence on the evolutionary and biogeographical patterns of European cyprinids. J Mol Evol.

[25] Machordom A, Doadrio I (2001). Evolutionary history and speciation modes in the cyprinid genus *Barbus*. Proc Biol Sci.

[26] Doadrio I, Carmona JA, Machordom A (2002). Haplotype diversity and phylogenetic relationships among the Iberian barbels (*Barbus*, Cyprinidae) reveal two evolutionary lineages. J Hered.

[27] Machordom A, Doadrio I (2001). Evidence of a cenozoic Betic-Kabilian connection based on freshwater fish phylogeography (*Luciobarbus*, Cyprinidae). Mol Phylogenet Evol.

[28] Mesquita N, Cunha C, Carvalho GR, Coelho MM. Comparative phylogeography of endemic cyprinids in the south-west Iberian Peninsula: evidence for a new ichthyogeographic area. J Fish Biol. 2007;71(sa).

[29] Burridge CP, Craw D, Jack DC, King TM, Waters JM (2008). Does fish ecology predict dispersal across a river drainage divide?. Evolution (N Y).

[30] Gante HF, Micael J, Oliva-Paterna FJ, Doadrio I, Dowling TE, Alves MJ (2009). Diversification within glacial refugia: tempo and mode of evolution of the polytypic fish *Barbus sclateri*. Mol Ecol.

[31] Doadrio I (1988). Sobre la taxonomía de *Barbus comiza* Steindachner, 1865 (Ostariophysi: Ciprinidae). Doñana Acta Vertebr.

[32] Filipe AF, Cowx IG, Collares-Pereira MJ (2002). Spatial modelling of freshwater fish in semi-arid river systems: a tool for conservation. River Res Appl.

[33] Filipe AF, Marques TA, Seabra S, Tiago P, Ribeiro F, Costa LMDA (2004). Selection of priority areas for fish conservation in Guadiana River Basin, Iberian Peninsula. Conserv Biol.

[34] Morán-López R, Pérez-Bote JL, Da Silva RE, Corbacho Amado C (2005). Summer habitat relationships of barbels in south-west Spain. J Fish Biol.

[35] Encina LE, Granado-Lorencio C (1989). A quantitative comparison of the jaw apparatus in three species of *Barbus* (Cyprinidae, Teleostei). J Anim Morphol Physiol.

[36] Encina LE, Granado-Lorencio C (1990). Morfoecologia trófica en el género *Barbus* (Pisces, Cyprinidae). Limnetica.

[37] Encina LE, Granado-Lorencio C (1997). Food habits and food resource partitioning in three coexisting *Barbus* species. Folia Zool.

[38] Pires AM, Cowx IG, Coelho MM (2001). Diet and growth of two sympatric Iberian barbel, *Barbus steindachneri* and *Barbus microcephalus*, in the middle reaches of the Guadiana Basin (Portugal). Folia Zool.

[39] Steindachner F (1866). Ichthyologischer Bericht über eine nach Spanien und Portugal unternommene Reise. (Fortsetzung) Über die Fische des Ebro und der Flusse bei Bilbao. Sitzungsb d kais Akad d Wissensehaft.

[40] Almaça C (1972). Sur la systématique des Barbeaux (genre et sous-genre *Barbus*) de la Péninsule Ibérique et de l’Afrique du Nord. Arq do Mus Bocage, 2a série.

[41] Almodóvar A, Nicola GG, Elvira B (2008). Natural hybridization of *Barbus bocagei* x *Barbus comizo* (Cyprinidae) in Tagus River basin, central Spain. Cybium.

[42] Callejas C, Ochando MD (2000). Recent radiation of Iberian barbel fish (Teleostei, Cyprinidae) inferred from cytochrome *b* genes. J Hered.

[43] Machordom A, Berrebi P, Doadrio I (1990). Spanish barbel hybridization detected using enzymatic markers: *Barbus meridionalis* Risso x *Barbus haasi* Mertens (Osteichthyes, Cyprinidae). Aquat Living Resour.

[44] Gante HF, Alves MJ, Dowling TE (2011). Paralog-specific primers for the amplification of nuclear loci in tetraploid barbels (*Barbus*: cypriniformes). J Hered.

[45] Gante HF, Alves MJ, Dowling TE (2008). Development of cytochrome *b* primers for mitotyping of barbels (Barbus spp.). Mol Ecol Resour.

[46] Machordom A, Doadrio I, Berrebi P (1995). Phylogeny and evolution of the genus Barbus in the Iberian Peninsula as revealed by allozyme electrophoresis. J Fish Biol.

[47] Tsigenopoulos CS, Durand JD, Ünlü E, Berrebi P (2003). Rapid radiation of the Mediterranean *Luciobarbus* species (Cyprinidae) after the Messinian salinity crisis of the Mediterranean Sea, inferred from mitochondrial phylogenetic analysis. Biol J Linn Soc.

[48] Evanno G, Regnaut S, Goudet J (2005). Detecting the number of clusters of individuals using the software STRUCTURE: a simulation study. Mol Ecol.

[49] Roe AD, Sperling FAH (2007). Population structure and species boundary delimitation of cryptic *Dioryctria* moths: an integrative approach. Mol Ecol.

[50] Markolf M, Rakotonirina H, Fichtel C, von Grumbkow P, Brameier M, Kappeler PM (2013). True lemurs…true species - species delimitation using multiple data sources in the brown lemur complex. BMC Evol Biol.

[51] Lecocq T, Dellicour S, Michez D, Lhomme P, Vanderplanck M, Valterová I (2013). Scent of a break-up: phylogeography and reproductive trait divergences in the red-tailed bumblebee (Bombus lapidarius). BMC Evol Biol.

[52] Bendik NF, Meik JM, Gluesenkamp AG, Roelke CE, Chippindale PT (2013). Biogeography, phylogeny, and morphological evolution of central Texas cave and spring salamanders. BMC Evol Biol.

[53] Thomé MTC, Zamudio KR, Haddad CFB, Alexandrino J (2012). Delimiting genetic units in Neotropical toads under incomplete lineage sorting and hybridization. BMC Evol Biol.

[54] Jacquemyn H, Brys R, Honnay O, Roldán-Ruiz I (2012). Asymmetric gene introgression in two closely related Orchis species: evidence from morphometric and genetic analyses. BMC Evol Biol.

[55] Kane NC, King MG, Barker MS, Raduski A, Karrenberg S, Yatabe Y (2009). Comparative genomic and population genetic analyses indicate highly porous genomes and high levels of gene flow between divergent Helianthus species. Evolution.

[56] Mims MC, Darrin Hulsey C, Fitzpatrick BM, Streelman JT (2010). Geography disentangles introgression from ancestral polymorphism in Lake Malawi cichlids. Mol Ecol.

[57] Martin SH, Dasmahapatra KK, Nadeau NJ, Salazar C, Walters JR, Simpson F (2013). Genome-wide evidence for speciation with gene flow in Heliconius butterflies. Genome Res.

[58] Peccoud J, Ollivier A, Plantegenest M, Simon J-C (2009). A continuum of genetic divergence from sympatric host races to species in the pea aphid complex. Proc Natl Acad Sci U S A.

[59] Wahlberg N, Weingartner E, Warren AD, Nylin S (2009). Timing major conflict between mitochondrial and nuclear genes in species relationships of Polygonia butterflies (Nymphalidae: Nymphalini). BMC Evol Biol.

[60] De Busschere C, Hendrickx F, Van Belleghem SM, Backeljau T, Lens L, Baert L (2010). Parallel habitat specialization within the wolf spider genus Hogna from the Galápagos. Mol Ecol.

[61] Fontenot BE, Makowsky R, Chippindale PT (2011). Nuclear-mitochondrial discordance and gene flow in a recent radiation of toads. Mol Phylogenet Evol.

[62] Pereira RJ, Monahan WB, Wake DB (2011). Predictors for reproductive isolation in a ring species complex following genetic and ecological divergence. BMC Evol Biol.

[63] Nadeau NJ, Martin SH, Kozak KM, Salazar C, Dasmahapatra KK, Davey JW (2013). Genome-wide patterns of divergence and gene flow across a butterfly radiation. Mol Ecol.

[64] Willis SC, Macrander J, Farias IP, Ortí G (2012). Simultaneous delimitation of species and quantification of interspecific hybridization in Amazonian peacock cichlids (genus Cichla) using multi-locus data. BMC Evol Biol.

[65] Gante HF, Santos CD, Alves MJ (2010). Phylogenetic relationships of the newly described species Chondrostoma olisiponensis (Teleostei: Cyprinidae). J Fish Biol.

[66] Sousa-Santos C, Gante HF, Robalo J, Proença Cunha P, Martins A, Arruda M (2014). Evolutionary history and population genetics of a cyprinid fish (Iberochondrostoma olisiponensis) endangered by introgression from a more abundant relative. Conserv Genet.

[67] Gante HF, Collares-Pereira MJ, Coelho MM (2004). Introgressive hybridisation between two Iberian Chondrostoma species (Teleostei, Cyprinidae) revisited: new evidence from morphology, mitochondrial DNA, allozymes and NOR-phenotypes. Folia Zool.

[68] Scribner KT, Page KS, Bartron ML (2000). Hybridization in freshwater species: a review of case studies and cytonuclear methods of biological inference. Rev Fish Biol Fish.

[69] Lajbner Z, Slechtová V, Slechta V, Svátora M, Berrebi P, Kotlík P (2009). Rare and asymmetrical hybridization of the endemic Barbus carpathicus with its widespread congener Barbus barbus. J Fish Biol.

[70] Meraner A, Venturi A, Ficetola GF, Rossi S, Candiotto A, Gandolfi A (2013). Massive invasion of exotic Barbus barbus and introgressive hybridization with endemic Barbus plebejus in Northern Italy: Where, how and why?. Mol Ecol.

[71] Pinho C, Hey J (2010). Divergence with gene flow: models and data. Annu Rev Ecol Evol Syst.

[72] Besansky NJ, Krzywinski J, Lehmann T, Simard F, Kern M, Mukabayire O (2003). Semipermeable species boundaries between Anopheles gambiae and Anopheles arabiensis: evidence from multilocus DNA sequence variation. Proc Natl Acad Sci U S A.

[73] Llopart A, Lachaise D, Coyne JA (2005). Multilocus analysis of introgression between two sympatric sister species of Drosophila: Drosophila yakuba and D. santomea. Genetics.

[74] Basset P, Yannic G, Brünner H, Hausser J (2006). Restricted gene flow at specific parts of the shrew genome in chromosomal hybrid zones. Evolution.

[75] Harr B (2006). Genomic islands of differentiation between house mouse subspecies. Genome Res.

[76] Carling MD, Brumfield RT (2009). Speciation in Passerina buntings: introgression patterns of sex-linked loci identify a candidate gene region for reproductive isolation. Mol Ecol.

[77] Carneiro M, Blanco-Aguiar JA, Villafuerte R, Ferrand N, Nachman MW (2010). Speciation in the European rabbit (Oryctolagus cuniculus): islands of differentiation on the X chromosome and autosomes. Evolution.

[78] Scascitelli M, Whitney KD, Randell RA, King M, Buerkle CA, Rieseberg LH (2010). Genome scan of hybridizing sunflowers from Texas (Helianthus annuus and H. debilis) reveals asymmetric patterns of introgression and small islands of genomic differentiation. Mol Ecol.

[79] Feder JL, Egan SP, Nosil P (2012). The genomics of speciation-with-gene-flow. Trends Genet.

[80] Kautt AF, Elmer KR, Meyer A (2012). Genomic signatures of divergent selection and speciation patterns in a “natural experiment”, the young parallel radiations of Nicaraguan crater lake cichlid fishes. Mol Ecol.

[81] Nadeau NJ, Whibley A, Jones RT, Davey JW, Dasmahapatra KK, Baxter SW (2012). Genomic islands of divergence in hybridizing Heliconius butterflies identified by large-scale targeted sequencing. Philos Trans R Soc Lond B Biol Sci.

[82] Feder JL, Nosil P (2010). The efficacy of divergence hitchhiking in generating genomic islands during ecological speciation. Evolution.

[83] Flaxman SM, Feder JL, Nosil P (2012). Spatially explicit models of divergence and genome hitchhiking. J Evol Biol.

[84] Flaxman SM, Wacholder AC, Feder JL, Nosil P: Theoretical models of the influence of genomic architecture on the dynamics of speciation. Mol Ecol 2014;23:4074–4088. 10.1111/mec.12750 24724861

[85] Tsigenopoulos CS, Kotlík P, Berrebi P (2002). Biogeography and pattern of gene flow among *Barbus* species (Teleostei: Cyprinidae) inhabiting the Italian Peninsula and neighbouring Adriatic drainages as revealed by allozyme and mitochondrial sequence data. Biol J Linn Soc.

[86] Lavin PA, McPhail JD (1986). Adaptive divergence of trophic phenotype among freshwater populations of the threespine stickleback (Gasterosteus aculeatus). Can J Fish Aquat Sci.

[87] Barlow GW (2000). The Cichlid Fishes: Nature’s Grand Experiment In Evolution.

[88] Mallet J (2007). Hybrid speciation. Nature.

[89] Douglas ME, Minckley WL, Tyus HM (1989). Qualitative characters, identification of Colorado River chubs (Cyprinidae: genus Gila) and the “art of seeing well”. Copeia.

[90] Banarescu P, Bogutskaya N (2003). The Freshwater Fishes of Europe Volume 5/II: Cyprinidae 2: Part II: Barbus.

[91] Saruwatari T, López JA, Pietsch TW (1997). Cyanine blue: a versatile and harmless stain for specimen observation. Copeia.

[92] Hammer Ø, Harper DAT, Ryan PD: PAST: Paleontological statistics software package for education and data analysis. Palaeontol Electron 2001, 4:http://palaeo-electronica.org/2001_1/past/issue1_01.htm.

[93] Kotlík P, Bogutskaya NG, Ekmekçi FG (2004). Circum Black Sea phylogeography of Barbus freshwater fishes: divergence in the Pontic glacial refugium. Mol Ecol.

[94] Flot J-F, Tillier A, Samadi S, Tillier S (2006). Phase determination from direct sequencing of length-variable DNA regions. Mol Ecol Notes.

[95] Flot J-F (2007). champuru 1.0: a computer software for unraveling mixtures of two DNA sequences of unequal lengths. Mol Ecol Notes.

[96] Stephens M, Smith NJ, Donnelly P (2001). A new statistical method for haplotype reconstruction from population data. Am J Hum Genet.

[97] Flot J-F (2010). seqphase: a web tool for interconverting phase input/output files and fasta sequence alignments. Mol Ecol Resour.

[98] Darriba D, Taboada GL, Doallo R, Posada D (2012). jModelTest 2: more models, new heuristics and parallel computing. Nat Methods.

[99] Guindon S, Gascuel O (2003). A simple, fast, and accurate algorithm to estimate large phylogenies by maximum likelihood. Syst Biol.

[100] Guindon S, Dufayard J-F, Lefort V, Anisimova M, Hordijk W, Gascuel O (2010). New algorithms and methods to estimate maximum-likelihood phylogenies: assessing the performance of PhyML 3.0. Syst Biol.

[101] Librado P, Rozas J (2009). DnaSP v5: a software for comprehensive analysis of DNA polymorphism data. Bioinformatics.

[102] Tamura K, Peterson D, Peterson N, Stecher G, Nei M, Kumar S (2011). MEGA5: molecular evolutionary genetics analysis using maximum likelihood, evolutionary distance, and maximum parsimony methods. Mol Biol Evol.

[103] Felsenstein J (2005). PHYLIP (Phylogeny Inference Package) version 3.6.

[104] Pritchard JK, Stephens M, Donnelly P (2000). Inference of population structure using multilocus genotype data. Genetics.

[105] Falush D, Stephens M, Pritchard JK (2003). Inference of population structure using multilocus genotype data: linked loci and correlated allele frequencies. Genetics.

[106] Maddison DR, Maddison WP (2003). MacClade 4: Analysis of phylogeny and character evolution.

[107] Pritchard JK, Wen W: Documentation for STRUCTURE software: Version 2. Available from http://pritch.bsd.uchicago.edu. 2003.

[108] Earl DA, vonHoldt BM (2011). STRUCTURE HARVESTER: a website and program for visualizing STRUCTURE output and implementing the Evanno method. Conserv Genet Resour.

[109] Jakobsson M, Rosenberg NA (2007). CLUMPP: a cluster matching and permutation program for dealing with label switching and multimodality in analysis of population structure. Bioinformatics.

[110] Rosenberg NA (2003). Distruct: a program for the graphical display of population structure. Mol Ecol Notes.

[111] Gante HF, Doadrio I, Alves MJ, Dowling TE: Data from: Semi-permeable species boundaries in Iberian barbels (Barbus and Luciobarbus, Cyprinidae). Dryad Digit Repos 2015:http://dx.doi.org/10.5061/dryad.m1572. 10.1186/s12862-015-0392-3 PMC4465174 26066794

