# Supplementary material for: Semi-permeable species boundaries in Iberian barbels (Barbus and Luciobarbus, Cyprinidae)
Source: BMC Evol Biol. 2015 Jun 12;15:111. doi: 10.1186/s12862-015-0392-3 (PMC4465174; doi:10.1186/s12862-015-0392-3)
Supplement: Additional file 1: — Divergence measures across population pairs of Iberian barbels. [file 12862_2015_392_MOESM1_ESM.pdf]

Additional file 1 – Divergence measures across population pairs of Iberian barbels

**mtDNA**				
Population 1	Population 2	D_a	D_{xy}	F_{ST}
L. bocagei Douro	*L. bocagei* Tejo	0.00000	0.00000	0.00000
L. bocagei Douro	*L. comizo* Tejo	0.01039	0.01299	0.80000
L. bocagei Douro	*L. steindachneri* Tejo	0.01455	0.01455	1.00000
L. bocagei Douro	*L. bocagei* Sado	0.00000	0.00000	0.00000
L. bocagei Douro	*L. comizo* Guadiana	0.01455	0.01455	1.00000
L. bocagei Douro	*L. steindachneri* Guadiana	0.01035	0.02056	0.50340
L. bocagei Douro	*L. microcephalus* Guadiana	0.05107	0.05247	0.97334
L. bocagei Douro	*L. sclateri* Guadiana	0.02116	0.03306	0.64000
L. bocagei Douro	*L. sclateri* Segura	0.03636	0.03636	1.00000
L. bocagei Douro	*L. graellsii* Ebro	0.04364	0.04364	1.00000
L. bocagei Douro	*L. guiraonis* Mijares	0.05455	0.05455	1.00000
L. bocagei Douro	*L. guiraonis* Júcar	0.06194	0.08400	0.73737
L. bocagei Douro	*B. haasi* Ebro	0.09818	0.09818	1.00000
L. bocagei Tejo	*L. comizo* Tejo	0.01039	0.01299	0.80000
L. bocagei Tejo	*L. steindachneri* Tejo	0.01455	0.01455	1.00000
L. bocagei Tejo	*L. bocagei* Sado	0.00000	0.00000	0.00000
L. bocagei Tejo	*L. comizo* Guadiana	0.01455	0.01455	1.00000
L. bocagei Tejo	*L. steindachneri* Guadiana	0.01035	0.02056	0.50340
L. bocagei Tejo	*L. microcephalus* Guadiana	0.05107	0.05247	0.97334
L. bocagei Tejo	*L. sclateri* Guadiana	0.02116	0.03306	0.64000
L. bocagei Tejo	*L. sclateri* Segura	0.03636	0.03636	1.00000
L. bocagei Tejo	*L. graellsii* Ebro	0.04364	0.04364	1.00000
L. bocagei Tejo	*L. guiraonis* Mijares	0.05455	0.05455	1.00000
L. bocagei Tejo	*L. guiraonis* Júcar	0.06194	0.08400	0.73737
L. bocagei Tejo	*B. haasi* Ebro	0.09818	0.09818	1.00000
L. comizo Tejo	*L. steindachneri* Tejo	0.00000	0.00260	0.00000
L. comizo Tejo	*L. bocagei* Sado	0.01039	0.01299	0.80000
L. comizo Tejo	*L. comizo* Guadiana	0.00000	0.00260	0.00000
L. comizo Tejo	*L. steindachneri* Guadiana	0.00156	0.01437	0.10848
L. comizo Tejo	*L. microcephalus* Guadiana	0.04810	0.05210	0.92330
L. comizo Tejo	*L. sclateri* Guadiana	0.02475	0.03924	0.63057
L. comizo Tejo	*L. sclateri* Segura	0.04675	0.04935	0.94737
L. comizo Tejo	*L. graellsii* Ebro	0.05403	0.05662	0.95413
L. comizo Tejo	*L. guiraonis* Mijares	0.05247	0.05506	0.95283
L. comizo Tejo	*L. guiraonis* Júcar	0.06423	0.08888	0.72258
L. comizo Tejo	*B. haasi* Ebro	0.10234	0.10494	0.97525
L. steindachneri Tejo	*L. bocagei* Sado	0.01455	0.01455	1.00000
L. steindachneri Tejo	*L. comizo* Guadiana	0.00000	0.00000	0.00000
L. steindachneri Tejo	*L. steindachneri* Guadiana	0.00252	0.01273	0.19780
L. steindachneri Tejo	*L. microcephalus* Guadiana	0.05003	0.05143	0.97281
L. steindachneri Tejo	*L. sclateri* Guadiana	0.02777	0.03967	0.70000

L. steindachneri Tejo	*L. sclateri* Segura	0.05091	0.05091	1.00000
L. steindachneri Tejo	*L. graellsii* Ebro	0.05818	0.05818	1.00000
L. steindachneri Tejo	*L. guiraonis* Mijares	0.05455	0.05455	1.00000
L. steindachneri Tejo	*L. guiraonis* Júcar	0.06703	0.08909	0.75238
L. steindachneri Tejo	*B. haasi* Ebro	0.10545	0.10545	1.00000
L. bocagei Sado	*L. comizo* Guadiana	0.01455	0.01455	1.00000
L. bocagei Sado	*L. steindachneri* Guadiana	0.01035	0.02056	0.50340
L. bocagei Sado	*L. microcephalus* Guadiana	0.05107	0.05247	0.97334
L. bocagei Sado	*L. sclateri* Guadiana	0.02116	0.03306	0.64000
L. bocagei Sado	*L. sclateri* Segura	0.03636	0.03636	1.00000
L. bocagei Sado	*L. graellsii* Ebro	0.04364	0.04364	1.00000
L. bocagei Sado	*L. guiraonis* Mijares	0.05455	0.05455	1.00000
L. bocagei Sado	*L. guiraonis* Júcar	0.06194	0.08400	0.73737
L. bocagei Sado	*B. haasi* Ebro	0.09818	0.09818	1.00000
L. comizo Guadiana	*L. steindachneri* Guadiana	0.00252	0.01273	0.19780
L. comizo Guadiana	*L. microcephalus* Guadiana	0.05003	0.05143	0.97281
L. comizo Guadiana	*L. sclateri* Guadiana	0.02777	0.03967	0.70000
L. comizo Guadiana	*L. sclateri* Segura	0.05091	0.05091	1.00000
L. comizo Guadiana	*L. graellsii* Ebro	0.05818	0.05818	1.00000
L. comizo Guadiana	*L. guiraonis* Mijares	0.05455	0.05455	1.00000
L. comizo Guadiana	*L. guiraonis* Júcar	0.06703	0.08909	0.75238
L. comizo Guadiana	*B. haasi* Ebro	0.10545	0.10545	1.00000
L. steindachneri Guadiana	*L. microcephalus* Guadiana	0.04188	0.05349	0.78297
L. steindachneri Guadiana	*L. sclateri* Guadiana	0.01198	0.03409	0.35136
L. steindachneri Guadiana	*L. sclateri* Segura	0.02993	0.04014	0.74564
L. steindachneri Guadiana	*L. graellsii* Ebro	0.04476	0.05497	0.81425
L. steindachneri Guadiana	*L. guiraonis* Mijares	0.04448	0.05469	0.81330
L. steindachneri Guadiana	*L. guiraonis* Júcar	0.05637	0.08864	0.63595
L. steindachneri Guadiana	*B. haasi* Ebro	0.09455	0.10476	0.90254
L. microcephalus Guadiana	*L. sclateri* Guadiana	0.04417	0.05747	0.76860
L. microcephalus Guadiana	*L. sclateri* Segura	0.05471	0.05610	0.97507
L. microcephalus Guadiana	*L. graellsii* Ebro	0.02198	0.02338	0.94017
L. microcephalus Guadiana	*L. guiraonis* Mijares	0.01107	0.01247	0.88782
L. microcephalus Guadiana	*L. guiraonis* Júcar	0.04610	0.06956	0.66274
L. microcephalus Guadiana	*B. haasi* Ebro	0.09419	0.09558	0.98537
L. sclateri Guadiana	*L. sclateri* Segura	0.00463	0.01653	0.28000
L. sclateri Guadiana	*L. graellsii* Ebro	0.03570	0.04760	0.75000
L. sclateri Guadiana	*L. guiraonis* Mijares	0.04264	0.05455	0.78182
L. sclateri Guadiana	*L. guiraonis* Júcar	0.05328	0.08724	0.61071
L. sclateri Guadiana	*B. haasi* Ebro	0.09091	0.10281	0.88424
L. sclateri Segura	*L. graellsii* Ebro	0.04000	0.04000	1.00000
L. sclateri Segura	*L. guiraonis* Mijares	0.05091	0.05091	1.00000
L. sclateri Segura	*L. guiraonis* Júcar	0.06085	0.08291	0.73392
L. sclateri Segura	*B. haasi* Ebro	0.09818	0.09818	1.00000

L. graellsii Ebro	*L. guiraonis* Mijares	0.02545	0.02545	1.00000
L. graellsii Ebro	*L. guiraonis* Júcar	0.04048	0.06255	0.64729
L. graellsii Ebro	*B. haasi* Ebro	0.08000	0.08000	1.00000
L. guiraonis Mijares	*L. guiraonis* Júcar	0.04776	0.06982	0.68403
L. guiraonis Mijares	*B. haasi* Ebro	0.09818	0.09818	1.00000
L. guiraonis Júcar	*B. haasi* Ebro	0.00630	0.02836	0.22222
**---**				
**All nuclear loci combined**				
Population 1	Population 2	D_a	D_{xy}	F_{ST}
L. bocagei Douro	*L. bocagei* Tejo	0.00029	0.00216	0.13286
L. bocagei Douro	*L. comizo* Tejo	0.00148	0.00354	0.41751
L. bocagei Douro	*L. steindachneri* Tejo	0.00071	0.00317	0.22299
L. bocagei Douro	*L. bocagei* Sado	0.00068	0.00233	0.29341
L. bocagei Douro	*L. comizo* Guadiana	0.00199	0.00417	0.47684
L. bocagei Douro	*L. steindachneri* Guadiana	0.00191	0.00468	0.40767
L. bocagei Douro	*L. microcephalus* Guadiana	0.00931	0.01099	0.84728
L. bocagei Douro	*L. sclateri* Guadiana	0.00359	0.00567	0.63238
L. bocagei Douro	*L. sclateri* Segura	0.00616	0.00776	0.79430
L. bocagei Douro	*L. graellsii* Ebro	0.01047	0.01223	0.85581
L. bocagei Douro	*L. guiraonis* Júcar	0.01683	0.02392	0.70382
L. bocagei Douro	*L. guiraonis* Mijares	0.01017	0.01148	0.88580
L. bocagei Douro	*H. haasi* Ebro	0.02830	0.02918	0.96967
L. bocagei Tejo	*L. comizo* Tejo	0.00123	0.00340	0.36151
L. bocagei Tejo	*L. steindachneri* Tejo	0.00042	0.00298	0.13933
L. bocagei Tejo	*L. bocagei* Sado	0.00027	0.00202	0.13350
L. bocagei Tejo	*L. comizo* Guadiana	0.00165	0.00393	0.41908
L. bocagei Tejo	*L. steindachneri* Guadiana	0.00155	0.00443	0.35020
L. bocagei Tejo	*L. microcephalus* Guadiana	0.00895	0.01073	0.83388
L. bocagei Tejo	*L. sclateri* Guadiana	0.00323	0.00541	0.59567
L. bocagei Tejo	*L. sclateri* Segura	0.00583	0.00753	0.77415
L. bocagei Tejo	*L. graellsii* Ebro	0.01010	0.01197	0.84390
L. bocagei Tejo	*L. guiraonis* Júcar	0.01646	0.02364	0.69598
L. bocagei Tejo	*L. guiraonis* Mijares	0.00981	0.01122	0.87380
L. bocagei Tejo	*H. haasi* Ebro	0.02792	0.02891	0.96576
L. comizo Tejo	*L. steindachneri* Tejo	0.00029	0.00304	0.09401
L. comizo Tejo	*L. bocagei* Sado	0.00126	0.00320	0.39376
L. comizo Tejo	*L. comizo* Guadiana	0.00060	0.00308	0.19573
L. comizo Tejo	*L. steindachneri* Guadiana	0.00079	0.00385	0.20495
L. comizo Tejo	*L. microcephalus* Guadiana	0.00888	0.01085	0.81842
L. comizo Tejo	*L. sclateri* Guadiana	0.00285	0.00522	0.54486
L. comizo Tejo	*L. sclateri* Segura	0.00554	0.00743	0.74566
L. comizo Tejo	*L. graellsii* Ebro	0.01005	0.01211	0.83014
L. comizo Tejo	*L. guiraonis* Júcar	0.01623	0.02361	0.68752

L. comizo Tejo	*L. guiraonis* Mijares	0.00976	0.01136	0.85877
L. comizo Tejo	*H. haasi* Ebro	0.02762	0.02879	0.95908
L. steindachneri Tejo	*L. bocagei* Sado	0.00052	0.00286	0.18055
L. steindachneri Tejo	*L. comizo* Guadiana	0.00048	0.00335	0.14219
L. steindachneri Tejo	*L. steindachneri* Guadiana	0.00055	0.00402	0.13783
L. steindachneri Tejo	*L. microcephalus* Guadiana	0.00847	0.01084	0.78142
L. steindachneri Tejo	*L. sclateri* Guadiana	0.00249	0.00527	0.47261
L. steindachneri Tejo	*L. sclateri* Segura	0.00515	0.00744	0.69237
L. steindachneri Tejo	*L. graellsii* Ebro	0.00964	0.01209	0.79693
L. steindachneri Tejo	*L. guiraonis* Júcar	0.01593	0.02370	0.67196
L. steindachneri Tejo	*L. guiraonis* Mijares	0.00934	0.01135	0.82341
L. steindachneri Tejo	*H. haasi* Ebro	0.02736	0.02894	0.94548
L. bocagei Sado	*L. comizo* Guadiana	0.00139	0.00345	0.40353
L. bocagei Sado	*L. steindachneri* Guadiana	0.00133	0.00398	0.33400
L. bocagei Sado	*L. microcephalus* Guadiana	0.00862	0.01018	0.84706
L. bocagei Sado	*L. sclateri* Guadiana	0.00301	0.00497	0.60495
L. bocagei Sado	*L. sclateri* Segura	0.00531	0.00678	0.78259
L. bocagei Sado	*L. graellsii* Ebro	0.00977	0.01142	0.85616
L. bocagei Sado	*L. guiraonis* Júcar	0.01614	0.02310	0.69864
L. bocagei Sado	*L. guiraonis* Mijares	0.00948	0.01067	0.88846
L. bocagei Sado	*H. haasi* Ebro	0.02761	0.02837	0.97307
L. comizo Guadiana	*L. steindachneri* Guadiana	0.00017	0.00335	0.05033
L. comizo Guadiana	*L. microcephalus* Guadiana	0.00844	0.01053	0.80170
L. comizo Guadiana	*L. sclateri* Guadiana	0.00236	0.00485	0.48595
L. comizo Guadiana	*L. sclateri* Segura	0.00503	0.00704	0.71493
L. comizo Guadiana	*L. graellsii* Ebro	0.00962	0.01179	0.81563
L. comizo Guadiana	*L. guiraonis* Júcar	0.01585	0.02335	0.67904
L. comizo Guadiana	*L. guiraonis* Mijares	0.00933	0.01105	0.84419
L. comizo Guadiana	*H. haasi* Ebro	0.02727	0.02856	0.95463
L. steindachneri Guadiana	*L. microcephalus* Guadiana	0.00803	0.01071	0.74987
L. steindachneri Guadiana	*L. sclateri* Guadiana	0.00110	0.00419	0.26279
L. steindachneri Guadiana	*L. sclateri* Segura	0.00350	0.00609	0.57382
L. steindachneri Guadiana	*L. graellsii* Ebro	0.00933	0.01209	0.77138
L. steindachneri Guadiana	*L. guiraonis* Júcar	0.01567	0.02376	0.65969
L. steindachneri Guadiana	*L. guiraonis* Mijares	0.00901	0.01132	0.79569
L. steindachneri Guadiana	*H. haasi* Ebro	0.02717	0.02906	0.93508
L. microcephalus Guadiana	*L. sclateri* Guadiana	0.00909	0.01109	0.82028
L. microcephalus Guadiana	*L. sclateri* Segura	0.01087	0.01238	0.87855
L. microcephalus Guadiana	*L. graellsii* Ebro	0.00308	0.00475	0.64861
L. microcephalus Guadiana	*L. guiraonis* Júcar	0.01433	0.02133	0.67218
L. microcephalus Guadiana	*L. guiraonis* Mijares	0.00241	0.00362	0.66374
L. microcephalus Guadiana	*H. haasi* Ebro	0.02850	0.02929	0.97294
L. sclateri Guadiana	*L. sclateri* Segura	0.00160	0.00351	0.45641
L. sclateri Guadiana	*L. graellsii* Ebro	0.01053	0.01261	0.83522

L. sclateri Guadiana	*L. guiraonis* Júcar	0.01701	0.02441	0.69689
L. sclateri Guadiana	*L. guiraonis* Mijares	0.01015	0.01178	0.86197
L. sclateri Guadiana	*H. haasi* Ebro	0.02863	0.02983	0.95978
L. sclateri Segura	*L. graellsii* Ebro	0.01224	0.01383	0.88515
L. sclateri Segura	*L. guiraonis* Júcar	0.01892	0.02583	0.73253
L. sclateri Segura	*L. guiraonis* Mijares	0.01195	0.01309	0.91315
L. sclateri Segura	*H. haasi* Ebro	0.03061	0.03132	0.97731
L. graellsii Ebro	*L. guiraonis* Júcar	0.01512	0.02219	0.68113
L. graellsii Ebro	*L. guiraonis* Mijares	0.00254	0.00385	0.66094
L. graellsii Ebro	*H. haasi* Ebro	0.02934	0.03022	0.97094
L. guiraonis Júcar	*L. guiraonis* Mijares	0.01445	0.02107	0.68566
L. guiraonis Júcar	*H. haasi* Ebro	0.00252	0.00872	0.28942
L. guiraonis Mijares	*H. haasi* Ebro	0.02896	0.02939	0.98549
--				
S7-1				
Population 1	Population 2	D_a	D_{xy}	F_{ST}
L. bocagei Douro	*L. bocagei* Tejo	0.00025	0.00238	0.10397
L. bocagei Douro	*L. comizo* Tejo	0.00043	0.00172	0.25160
L. bocagei Douro	*L. steindachneri* Tejo	0.00034	0.00217	0.15843
L. bocagei Douro	*L. bocagei* Sado	0.00071	0.00238	0.29927
L. bocagei Douro	*L. comizo* Guadiana	0.00064	0.00191	0.33414
L. bocagei Douro	*L. steindachneri* Guadiana	0.00099	0.00384	0.25765
L. bocagei Douro	*L. microcephalus* Guadiana	0.01428	0.01650	0.86521
L. bocagei Douro	*L. sclateri* Guadiana	0.00469	0.00700	0.67005
L. bocagei Douro	*L. sclateri* Segura	0.00756	0.00846	0.89343
L. bocagei Douro	*L. graellsii* Ebro	0.01219	0.01406	0.86724
L. bocagei Douro	*L. guiraonis* Júcar	0.01245	0.02126	0.58587
L. bocagei Douro	*L. guiraonis* Mijares	0.01102	0.01266	0.87075
L. bocagei Douro	*H. haasi* Ebro	0.02714	0.02804	0.96784
L. bocagei Tejo	*L. comizo* Tejo	0.00029	0.00191	0.15122
L. bocagei Tejo	*L. steindachneri* Tejo	0.00008	0.00224	0.03513
L. bocagei Tejo	*L. bocagei* Sado	0.00040	0.00240	0.16568
L. bocagei Tejo	*L. comizo* Guadiana	0.00061	0.00221	0.27700
L. bocagei Tejo	*L. steindachneri* Guadiana	0.00096	0.00414	0.23199
L. bocagei Tejo	*L. microcephalus* Guadiana	0.01428	0.01684	0.84816
L. bocagei Tejo	*L. sclateri* Guadiana	0.00469	0.00733	0.63946
L. bocagei Tejo	*L. sclateri* Segura	0.00757	0.00880	0.85978
L. bocagei Tejo	*L. graellsii* Ebro	0.01220	0.01439	0.84728
L. bocagei Tejo	*L. guiraonis* Júcar	0.01246	0.02160	0.57697
L. bocagei Tejo	*L. guiraonis* Mijares	0.01103	0.01300	0.84855
L. bocagei Tejo	*H. haasi* Ebro	0.02715	0.02838	0.95652
L. comizo Tejo	*L. steindachneri* Tejo	0.00004	0.00135	0.02786
L. comizo Tejo	*L. bocagei* Sado	0.00033	0.00148	0.21985

L. comizo Tejo	*L. comizo* Guadiana	0.00009	0.00084	0.10195
L. comizo Tejo	*L. steindachneri* Guadiana	0.00044	0.00277	0.15787
L. comizo Tejo	*L. microcephalus* Guadiana	0.01373	0.01543	0.88941
L. comizo Tejo	*L. sclateri* Guadiana	0.00414	0.00593	0.69784
L. comizo Tejo	*L. sclateri* Segura	0.00701	0.00739	0.94802
L. comizo Tejo	*L. graellsii* Ebro	0.01164	0.01299	0.89616
L. comizo Tejo	*L. guiraonis* Júcar	0.01190	0.02019	0.58958
L. comizo Tejo	*L. guiraonis* Mijares	0.01047	0.01159	0.90348
L. comizo Tejo	*H. haasi* Ebro	0.02659	0.02697	0.98576
L. steindachneri Tejo	*L. bocagei* Sado	0.00011	0.00180	0.05940
L. steindachneri Tejo	*L. comizo* Guadiana	0.00024	0.00154	0.15754
L. steindachneri Tejo	*L. steindachneri* Guadiana	0.00059	0.00346	0.16938
L. steindachneri Tejo	*L. microcephalus* Guadiana	0.01393	0.01618	0.86094
L. steindachneri Tejo	*L. sclateri* Guadiana	0.00433	0.00666	0.64955
L. steindachneri Tejo	*L. sclateri* Segura	0.00722	0.00814	0.88616
L. steindachneri Tejo	*L. graellsii* Ebro	0.01185	0.01374	0.86232
L. steindachneri Tejo	*L. guiraonis* Júcar	0.01211	0.02094	0.57835
L. steindachneri Tejo	*L. guiraonis* Mijares	0.01068	0.01234	0.86536
L. steindachneri Tejo	*H. haasi* Ebro	0.02680	0.02772	0.96657
L. bocagei Sado	*L. comizo* Guadiana	0.00059	0.00173	0.34343
L. bocagei Sado	*L. steindachneri* Guadiana	0.00094	0.00366	0.25730
L. bocagei Sado	*L. microcephalus* Guadiana	0.01427	0.01636	0.87212
L. bocagei Sado	*L. sclateri* Guadiana	0.00467	0.00685	0.68205
L. bocagei Sado	*L. sclateri* Segura	0.00755	0.00832	0.90756
L. bocagei Sado	*L. graellsii* Ebro	0.01218	0.01392	0.87543
L. bocagei Sado	*L. guiraonis* Júcar	0.01245	0.02112	0.58940
L. bocagei Sado	*L. guiraonis* Mijares	0.01101	0.01252	0.87989
L. bocagei Sado	*H. haasi* Ebro	0.02713	0.02790	0.97243
L. comizo Guadiana	*L. steindachneri* Guadiana	0.00028	0.00260	0.10898
L. comizo Guadiana	*L. microcephalus* Guadiana	0.01376	0.01545	0.89060
L. comizo Guadiana	*L. sclateri* Guadiana	0.00413	0.00590	0.69940
L. comizo Guadiana	*L. sclateri* Segura	0.00706	0.00743	0.95059
L. comizo Guadiana	*L. graellsii* Ebro	0.01169	0.01302	0.89777
L. comizo Guadiana	*L. guiraonis* Júcar	0.01196	0.02023	0.59118
L. comizo Guadiana	*L. guiraonis* Mijares	0.01052	0.01163	0.90526
L. comizo Guadiana	*H. haasi* Ebro	0.02664	0.02701	0.98641
L. steindachneri Guadiana	*L. microcephalus* Guadiana	0.01269	0.01596	0.79522
L. steindachneri Guadiana	*L. sclateri* Guadiana	0.00212	0.00548	0.38764
L. steindachneri Guadiana	*L. sclateri* Segura	0.00438	0.00632	0.69220
L. steindachneri Guadiana	*L. graellsii* Ebro	0.01102	0.01393	0.79117
L. steindachneri Guadiana	*L. guiraonis* Júcar	0.01134	0.02118	0.53516
L. steindachneri Guadiana	*L. guiraonis* Mijares	0.00971	0.01239	0.78373
L. steindachneri Guadiana	*H. haasi* Ebro	0.02624	0.02819	0.93098
L. microcephalus Guadiana	*L. sclateri* Guadiana	0.01470	0.01743	0.84335

L. microcephalus Guadiana	*L. sclateri* Segura	0.01666	0.01798	0.92644
L. microcephalus Guadiana	*L. graellsii* Ebro	0.00605	0.00834	0.72566
L. microcephalus Guadiana	*L. guiraonis* Júcar	0.01171	0.02093	0.55934
L. microcephalus Guadiana	*L. guiraonis* Mijares	0.00377	0.00583	0.64689
L. microcephalus Guadiana	*H. haasi* Ebro	0.03219	0.03352	0.96053
L. sclateri Guadiana	*L. sclateri* Segura	0.00031	0.00172	0.17989
L. sclateri Guadiana	*L. graellsii* Ebro	0.01342	0.01579	0.84982
L. sclateri Guadiana	*L. guiraonis* Júcar	0.01347	0.02278	0.59134
L. sclateri Guadiana	*L. guiraonis* Mijares	0.01181	0.01395	0.84646
L. sclateri Guadiana	*H. haasi* Ebro	0.02854	0.02994	0.95299
L. sclateri Segura	*L. graellsii* Ebro	0.01561	0.01657	0.94182
L. sclateri Segura	*L. guiraonis* Júcar	0.01559	0.02350	0.66369
L. sclateri Segura	*L. guiraonis* Mijares	0.01386	0.01460	0.94970
L. sclateri Segura	*H. haasi* Ebro	0.03077	0.03077	1.00000
L. graellsii Ebro	*L. guiraonis* Júcar	0.00969	0.01855	0.52209
L. graellsii Ebro	*L. guiraonis* Mijares	0.00202	0.00372	0.54279
L. graellsii Ebro	*H. haasi* Ebro	0.02980	0.03077	0.96866
L. guiraonis Júcar	*L. guiraonis* Mijares	0.00830	0.01694	0.49020
L. guiraonis Júcar	*H. haasi* Ebro	0.00545	0.01336	0.40838
L. guiraonis Mijares	*H. haasi* Ebro	0.02864	0.02937	0.97500
**---**				
S7-2				
Population 1	Population 2	D_a	D_{xy}	F_{ST}
L. bocagei Douro	*L. bocagei* Tejo	0.00056	0.00311	0.18021
L. bocagei Douro	*L. comizo* Tejo	0.00064	0.00438	0.14634
L. bocagei Douro	*L. steindachneri* Tejo	0.00088	0.00416	0.21231
L. bocagei Douro	*L. bocagei* Sado	0.00169	0.00415	0.40630
L. bocagei Douro	*L. comizo* Guadiana	0.00358	0.00641	0.55841
L. bocagei Douro	*L. steindachneri* Guadiana	0.00283	0.00627	0.45107
L. bocagei Douro	*L. microcephalus* Guadiana	0.00870	0.01058	0.82206
L. bocagei Douro	*L. sclateri* Guadiana	0.00405	0.00622	0.65074
L. bocagei Douro	*L. sclateri* Segura	0.00687	0.00884	0.77706
L. bocagei Douro	*L. graellsii* Ebro	0.01089	0.01343	0.81058
L. bocagei Douro	*L. guiraonis* Júcar	0.01417	0.01875	0.75580
L. bocagei Douro	*L. guiraonis* Mijares	0.00836	0.00955	0.87535
L. bocagei Douro	*H. haasi* Ebro	0.01986	0.02105	0.94342
L. bocagei Tejo	*L. comizo* Tejo	0.00060	0.00451	0.13336
L. bocagei Tejo	*L. steindachneri* Tejo	0.00039	0.00383	0.10241
L. bocagei Tejo	*L. bocagei* Sado	0.00075	0.00338	0.22099
L. bocagei Tejo	*L. comizo* Guadiana	0.00265	0.00564	0.46894
L. bocagei Tejo	*L. steindachneri* Guadiana	0.00165	0.00526	0.31451
L. bocagei Tejo	*L. microcephalus* Guadiana	0.00790	0.00995	0.79411
L. bocagei Tejo	*L. sclateri* Guadiana	0.00256	0.00490	0.52304

L. bocagei Tejo	*L. sclateri* Segura	0.00535	0.00748	0.71470
L. bocagei Tejo	*L. graellsii* Ebro	0.01009	0.01280	0.78829
L. bocagei Tejo	*L. guiraonis* Júcar	0.01337	0.01812	0.73813
L. bocagei Tejo	*L. guiraonis* Mijares	0.00756	0.00892	0.84797
L. bocagei Tejo	*H. haasi* Ebro	0.01906	0.02041	0.93357
L. comizo Tejo	*L. steindachneri* Tejo	0.00007	0.00471	0.01559
L. comizo Tejo	*L. bocagei* Sado	0.00092	0.00474	0.19346
L. comizo Tejo	*L. comizo* Guadiana	0.00124	0.00543	0.22781
L. comizo Tejo	*L. steindachneri* Guadiana	0.00112	0.00592	0.18950
L. comizo Tejo	*L. microcephalus* Guadiana	0.00753	0.01078	0.69917
L. comizo Tejo	*L. sclateri* Guadiana	0.00301	0.00654	0.45986
L. comizo Tejo	*L. sclateri* Segura	0.00591	0.00924	0.63960
L. comizo Tejo	*L. graellsii* Ebro	0.00973	0.01363	0.71359
L. comizo Tejo	*L. guiraonis* Júcar	0.01301	0.01894	0.68658
L. comizo Tejo	*L. guiraonis* Mijares	0.00720	0.00975	0.73846
L. comizo Tejo	*H. haasi* Ebro	0.01869	0.02124	0.87997
L. steindachneri Tejo	*L. bocagei* Sado	0.00069	0.00404	0.16974
L. steindachneri Tejo	*L. comizo* Guadiana	0.00101	0.00473	0.21345
L. steindachneri Tejo	*L. steindachneri* Guadiana	0.00073	0.00506	0.14462
L. steindachneri Tejo	*L. microcephalus* Guadiana	0.00749	0.01026	0.72963
L. steindachneri Tejo	*L. sclateri* Guadiana	0.00254	0.00561	0.45326
L. steindachneri Tejo	*L. sclateri* Segura	0.00545	0.00831	0.65573
L. steindachneri Tejo	*L. graellsii* Ebro	0.00968	0.01312	0.73798
L. steindachneri Tejo	*L. guiraonis* Júcar	0.01296	0.01843	0.70319
L. steindachneri Tejo	*L. guiraonis* Mijares	0.00715	0.00924	0.77449
L. steindachneri Tejo	*H. haasi* Ebro	0.01865	0.02073	0.89952
L. bocagei Sado	*L. comizo* Guadiana	0.00169	0.00461	0.36733
L. bocagei Sado	*L. steindachneri* Guadiana	0.00095	0.00447	0.21194
L. bocagei Sado	*L. microcephalus* Guadiana	0.00689	0.00886	0.77802
L. bocagei Sado	*L. sclateri* Guadiana	0.00211	0.00436	0.48266
L. bocagei Sado	*L. sclateri* Segura	0.00369	0.00574	0.64232
L. bocagei Sado	*L. graellsii* Ebro	0.00908	0.01171	0.77559
L. bocagei Sado	*L. guiraonis* Júcar	0.01236	0.01703	0.72618
L. bocagei Sado	*L. guiraonis* Mijares	0.00656	0.00783	0.83728
L. bocagei Sado	*H. haasi* Ebro	0.01805	0.01932	0.93406
L. comizo Guadiana	*L. steindachneri* Guadiana	0.00043	0.00432	0.10058
L. comizo Guadiana	*L. microcephalus* Guadiana	0.00731	0.00965	0.75825
L. comizo Guadiana	*L. sclateri* Guadiana	0.00332	0.00594	0.55863
L. comizo Guadiana	*L. sclateri* Segura	0.00638	0.00880	0.72510
L. comizo Guadiana	*L. graellsii* Ebro	0.00951	0.01250	0.76049
L. comizo Guadiana	*L. guiraonis* Júcar	0.01272	0.01774	0.71663
L. comizo Guadiana	*L. guiraonis* Mijares	0.00698	0.00862	0.80972
L. comizo Guadiana	*H. haasi* Ebro	0.01838	0.02003	0.91809
L. steindachneri Guadiana	*L. microcephalus* Guadiana	0.00704	0.00998	0.70527

L. steindachneri Guadiana	*L. sclateri* Guadiana	0.00119	0.00442	0.26859
L. steindachneri Guadiana	*L. sclateri* Segura	0.00398	0.00700	0.56773
L. steindachneri Guadiana	*L. graellsii* Ebro	0.00931	0.01291	0.72105
L. steindachneri Guadiana	*L. guiraonis* Júcar	0.01257	0.01821	0.69044
L. steindachneri Guadiana	*L. guiraonis* Mijares	0.00679	0.00904	0.75109
L. steindachneri Guadiana	*H. haasi* Ebro	0.01825	0.02050	0.89031
L. microcephalus Guadiana	*L. sclateri* Guadiana	0.00864	0.01032	0.83768
L. microcephalus Guadiana	*L. sclateri* Segura	0.01151	0.01298	0.88672
L. microcephalus Guadiana	*L. graellsii* Ebro	0.00286	0.00491	0.58305
L. microcephalus Guadiana	*L. guiraonis* Júcar	0.01304	0.01712	0.76168
L. microcephalus Guadiana	*L. guiraonis* Mijares	0.00033	0.00103	0.32593
L. microcephalus Guadiana	*H. haasi* Ebro	0.02045	0.02114	0.96728
L. sclateri Guadiana	*L. sclateri* Segura	0.00239	0.00415	0.57521
L. sclateri Guadiana	*L. graellsii* Ebro	0.01108	0.01341	0.82583
L. sclateri Guadiana	*L. guiraonis* Júcar	0.01436	0.01873	0.76666
L. sclateri Guadiana	*L. guiraonis* Mijares	0.00855	0.00954	0.89693
L. sclateri Guadiana	*H. haasi* Ebro	0.02005	0.02103	0.95327
L. sclateri Segura	*L. graellsii* Ebro	0.01396	0.01609	0.86748
L. sclateri Segura	*L. guiraonis* Júcar	0.01724	0.02141	0.80537
L. sclateri Segura	*L. guiraonis* Mijares	0.01143	0.01221	0.93622
L. sclateri Segura	*H. haasi* Ebro	0.02293	0.02371	0.96715
L. graellsii Ebro	*L. guiraonis* Júcar	0.01523	0.01997	0.76259
L. graellsii Ebro	*L. guiraonis* Mijares	0.00253	0.00388	0.65107
L. graellsii Ebro	*H. haasi* Ebro	0.02264	0.02399	0.94359
L. guiraonis Júcar	*L. guiraonis* Mijares	0.01270	0.01609	0.78947
L. guiraonis Júcar	*H. haasi* Ebro	0.00064	0.00402	0.15789
L. guiraonis Mijares	*H. haasi* Ebro	0.02011	0.02011	1.00000
--				
Gh-1				
Population 1	Population 2	D_a	D_{xy}	F_{ST}
L. bocagei Douro	*L. bocagei* Tejo	0.00001	0.00019	0.02899
L. bocagei Douro	*L. comizo* Tejo	0.00135	0.00206	0.65587
L. bocagei Douro	*L. steindachneri* Tejo	0.00051	0.00135	0.37630
L. bocagei Douro	*L. bocagei* Sado	0.00000	0.00008	0.00000
L. bocagei Douro	*L. comizo* Guadiana	0.00178	0.00227	0.78333
L. bocagei Douro	*L. steindachneri* Guadiana	0.00167	0.00233	0.71814
L. bocagei Douro	*L. microcephalus* Guadiana	0.00152	0.00152	1.00000
L. bocagei Douro	*L. sclateri* Guadiana	0.00169	0.00248	0.68254
L. bocagei Douro	*L. sclateri* Segura	0.00204	0.00242	0.84211
L. bocagei Douro	*L. graellsii* Ebro	0.00343	0.00394	0.87045
L. bocagei Douro	*L. guiraonis* Júcar	0.01601	0.02068	0.77424
L. bocagei Douro	*L. guiraonis* Mijares	0.00525	0.00606	0.86667
L. bocagei Douro	*H. haasi* Ebro	0.02424	0.02424	1.00000

L. bocagei Tejo	*L. comizo* Tejo	0.00135	0.00225	0.60300
L. bocagei Tejo	*L. steindachneri* Tejo	0.00051	0.00154	0.33364
L. bocagei Tejo	*L. bocagei* Sado	0.00001	0.00027	0.02070
L. bocagei Tejo	*L. comizo* Guadiana	0.00179	0.00246	0.72531
L. bocagei Tejo	*L. steindachneri* Guadiana	0.00168	0.00252	0.66635
L. bocagei Tejo	*L. microcephalus* Guadiana	0.00152	0.00170	0.89211
L. bocagei Tejo	*L. sclateri* Guadiana	0.00170	0.00267	0.63616
L. bocagei Tejo	*L. sclateri* Segura	0.00205	0.00261	0.78318
L. bocagei Tejo	*L. graellsii* Ebro	0.00343	0.00413	0.83185
L. bocagei Tejo	*L. guiraonis* Júcar	0.01602	0.02087	0.76748
L. bocagei Tejo	*L. guiraonis* Mijares	0.00526	0.00625	0.84128
L. bocagei Tejo	*H. haasi* Ebro	0.02425	0.02443	0.99247
L. comizo Tejo	*L. steindachneri* Tejo	0.00021	0.00176	0.11980
L. comizo Tejo	*L. bocagei* Sado	0.00124	0.00202	0.61292
L. comizo Tejo	*L. comizo* Guadiana	0.00084	0.00204	0.41253
L. comizo Tejo	*L. steindachneri* Guadiana	0.00082	0.00219	0.37554
L. comizo Tejo	*L. microcephalus* Guadiana	0.00070	0.00141	0.49704
L. comizo Tejo	*L. sclateri* Guadiana	0.00088	0.00237	0.36961
L. comizo Tejo	*L. sclateri* Segura	0.00123	0.00232	0.52919
L. comizo Tejo	*L. graellsii* Ebro	0.00261	0.00383	0.68208
L. comizo Tejo	*L. guiraonis* Júcar	0.01520	0.02057	0.73866
L. comizo Tejo	*L. guiraonis* Mijares	0.00444	0.00595	0.74536
L. comizo Tejo	*H. haasi* Ebro	0.02343	0.02413	0.97068
L. steindachneri Tejo	*L. bocagei* Sado	0.00043	0.00135	0.31757
L. steindachneri Tejo	*L. comizo* Guadiana	0.00062	0.00195	0.31647
L. steindachneri Tejo	*L. steindachneri* Guadiana	0.00050	0.00200	0.25120
L. steindachneri Tejo	*L. microcephalus* Guadiana	0.00040	0.00124	0.32206
L. steindachneri Tejo	*L. sclateri* Guadiana	0.00051	0.00214	0.23789
L. steindachneri Tejo	*L. sclateri* Segura	0.00093	0.00215	0.43050
L. steindachneri Tejo	*L. graellsii* Ebro	0.00231	0.00367	0.63091
L. steindachneri Tejo	*L. guiraonis* Júcar	0.01490	0.02041	0.72991
L. steindachneri Tejo	*L. guiraonis* Mijares	0.00414	0.00579	0.71471
L. steindachneri Tejo	*H. haasi* Ebro	0.02313	0.02397	0.96480
L. bocagei Sado	*L. comizo* Guadiana	0.00163	0.00220	0.74138
L. bocagei Sado	*L. steindachneri* Guadiana	0.00152	0.00226	0.67508
L. bocagei Sado	*L. microcephalus* Guadiana	0.00136	0.00144	0.94737
L. bocagei Sado	*L. sclateri* Guadiana	0.00154	0.00240	0.64102
L. bocagei Sado	*L. sclateri* Segura	0.00189	0.00235	0.80475
L. bocagei Sado	*L. graellsii* Ebro	0.00328	0.00386	0.84830
L. bocagei Sado	*L. guiraonis* Júcar	0.01586	0.02061	0.76974
L. bocagei Sado	*L. guiraonis* Mijares	0.00510	0.00598	0.85232
L. bocagei Sado	*H. haasi* Ebro	0.02409	0.02417	0.99687
L. comizo Guadiana	*L. steindachneri* Guadiana	0.00006	0.00121	0.05228
L. comizo Guadiana	*L. microcephalus* Guadiana	0.00027	0.00076	0.35000

L. comizo Guadiana	*L. sclateri* Guadiana	0.00044	0.00172	0.25686
L. comizo Guadiana	*L. sclateri* Segura	0.00079	0.00167	0.47488
L. comizo Guadiana	*L. graellsii* Ebro	0.00218	0.00318	0.68484
L. comizo Guadiana	*L. guiraonis* Júcar	0.01476	0.01992	0.74094
L. comizo Guadiana	*L. guiraonis* Mijares	0.00400	0.00530	0.75476
L. comizo Guadiana	*H. haasi* Ebro	0.02299	0.02348	0.97903
L. steindachneri Guadiana	*L. microcephalus* Guadiana	0.00016	0.00082	0.19468
L. steindachneri Guadiana	*L. sclateri* Guadiana	0.00014	0.00159	0.08985
L. steindachneri Guadiana	*L. sclateri* Segura	0.00069	0.00172	0.39720
L. steindachneri Guadiana	*L. graellsii* Ebro	0.00207	0.00324	0.63970
L. steindachneri Guadiana	*L. guiraonis* Júcar	0.01466	0.01998	0.73346
L. steindachneri Guadiana	*L. guiraonis* Mijares	0.00390	0.00536	0.72673
L. steindachneri Guadiana	*H. haasi* Ebro	0.02289	0.02354	0.97209
L. microcephalus Guadiana	*L. sclateri* Guadiana	0.00018	0.00096	0.18367
L. microcephalus Guadiana	*L. sclateri* Segura	0.00053	0.00091	0.57895
L. microcephalus Guadiana	*L. graellsii* Ebro	0.00191	0.00242	0.78947
L. microcephalus Guadiana	*L. guiraonis* Júcar	0.01450	0.01917	0.75640
L. microcephalus Guadiana	*L. guiraonis* Mijares	0.00374	0.00455	0.82222
L. microcephalus Guadiana	*H. haasi* Ebro	0.02273	0.02273	1.00000
L. sclateri Guadiana	*L. sclateri* Segura	0.00070	0.00187	0.37550
L. sclateri Guadiana	*L. graellsii* Ebro	0.00209	0.00339	0.61709
L. sclateri Guadiana	*L. guiraonis* Júcar	0.01467	0.02013	0.72897
L. sclateri Guadiana	*L. guiraonis* Mijares	0.00391	0.00551	0.71048
L. sclateri Guadiana	*H. haasi* Ebro	0.02290	0.02369	0.96678
L. sclateri Segura	*L. graellsii* Ebro	0.00244	0.00333	0.73206
L. sclateri Segura	*L. guiraonis* Júcar	0.01502	0.02008	0.74836
L. sclateri Segura	*L. guiraonis* Mijares	0.00426	0.00545	0.78168
L. sclateri Segura	*H. haasi* Ebro	0.02325	0.02364	0.98381
L. graellsii Ebro	*L. guiraonis* Júcar	0.01544	0.02062	0.74883
L. graellsii Ebro	*L. guiraonis* Mijares	0.00080	0.00212	0.37845
L. graellsii Ebro	*H. haasi* Ebro	0.02464	0.02515	0.97971
L. guiraonis Júcar	*L. guiraonis* Mijares	0.01664	0.02212	0.75240
L. guiraonis Júcar	*H. haasi* Ebro	0.00086	0.00553	0.15573
L. guiraonis Mijares	*H. haasi* Ebro	0.02646	0.02727	0.97037
**---**				
Gh-2				
Population 1	Population 2	D_a	D_{xy}	F_{ST}
L. bocagei Douro	*L. bocagei* Tejo	0.00032	0.00271	0.11605
L. bocagei Douro	*L. comizo* Tejo	0.00309	0.00547	0.56423
L. bocagei Douro	*L. steindachneri* Tejo	0.00101	0.00456	0.22158
L. bocagei Douro	*L. bocagei* Sado	0.00038	0.00255	0.15016
L. bocagei Douro	*L. comizo* Guadiana	0.00198	0.00565	0.35050
L. bocagei Douro	*L. steindachneri* Guadiana	0.00210	0.00587	0.35825

L. bocagei Douro	*L. microcephalus* Guadiana	0.01160	0.01392	0.83272
L. bocagei Douro	*L. sclateri* Guadiana	0.00374	0.00655	0.57168
L. bocagei Douro	*L. sclateri* Segura	0.00756	0.01033	0.73164
L. bocagei Douro	*L. graellsii* Ebro	0.01400	0.01600	0.87495
L. bocagei Douro	*L. guiraonis* Júcar	0.02310	0.03258	0.70915
L. bocagei Douro	*L. guiraonis* Mijares	0.01460	0.01612	0.90567
L. bocagei Douro	*H. haasi* Ebro	0.03894	0.04024	0.96786
L. bocagei Tejo	*L. comizo* Tejo	0.00239	0.00459	0.52095
L. bocagei Tejo	*L. steindachneri* Tejo	0.00063	0.00400	0.15851
L. bocagei Tejo	*L. bocagei* Sado	-0.00001	0.00197	-0.00561
L. bocagei Tejo	*L. comizo* Guadiana	0.00160	0.00508	0.31450
L. bocagei Tejo	*L. steindachneri* Guadiana	0.00185	0.00543	0.34072
L. bocagei Tejo	*L. microcephalus* Guadiana	0.01101	0.01315	0.83715
L. bocagei Tejo	*L. sclateri* Guadiana	0.00371	0.00632	0.58618
L. bocagei Tejo	*L. sclateri* Segura	0.00763	0.01022	0.74697
L. bocagei Tejo	*L. graellsii* Ebro	0.01340	0.01521	0.88072
L. bocagei Tejo	*L. guiraonis* Júcar	0.02246	0.03175	0.70746
L. bocagei Tejo	*L. guiraonis* Mijares	0.01399	0.01532	0.91299
L. bocagei Tejo	*H. haasi* Ebro	0.03829	0.03940	0.97192
L. comizo Tejo	*L. steindachneri* Tejo	0.00071	0.00406	0.17528
L. comizo Tejo	*L. bocagei* Sado	0.00231	0.00427	0.54043
L. comizo Tejo	*L. comizo* Guadiana	0.00034	0.00381	0.08929
L. comizo Tejo	*L. steindachneri* Guadiana	0.00079	0.00436	0.18168
L. comizo Tejo	*L. microcephalus* Guadiana	0.01215	0.01428	0.85097
L. comizo Tejo	*L. sclateri* Guadiana	0.00315	0.00575	0.54734
L. comizo Tejo	*L. sclateri* Segura	0.00728	0.00985	0.73911
L. comizo Tejo	*L. graellsii* Ebro	0.01459	0.01639	0.89019
L. comizo Tejo	*L. guiraonis* Júcar	0.02306	0.03233	0.71321
L. comizo Tejo	*L. guiraonis* Mijares	0.01518	0.01650	0.92009
L. comizo Tejo	*H. haasi* Ebro	0.03864	0.03973	0.97253
L. steindachneri Tejo	*L. bocagei* Sado	0.00078	0.00391	0.19935
L. steindachneri Tejo	*L. comizo* Guadiana	0.00014	0.00477	0.02887
L. steindachneri Tejo	*L. steindachneri* Guadiana	0.00042	0.00515	0.08236
L. steindachneri Tejo	*L. microcephalus* Guadiana	0.01088	0.01417	0.76760
L. steindachneri Tejo	*L. sclateri* Guadiana	0.00244	0.00621	0.39291
L. steindachneri Tejo	*L. sclateri* Segura	0.00640	0.01013	0.63144
L. steindachneri Tejo	*L. graellsii* Ebro	0.01330	0.01626	0.81772
L. steindachneri Tejo	*L. guiraonis* Júcar	0.02214	0.03258	0.67962
L. steindachneri Tejo	*L. guiraonis* Mijares	0.01389	0.01638	0.84835
L. steindachneri Tejo	*H. haasi* Ebro	0.03788	0.04013	0.94377
L. bocagei Sado	*L. comizo* Guadiana	0.00163	0.00488	0.33389
L. bocagei Sado	*L. steindachneri* Guadiana	0.00180	0.00515	0.34999
L. bocagei Sado	*L. microcephalus* Guadiana	0.01085	0.01275	0.85038
L. bocagei Sado	*L. sclateri* Guadiana	0.00347	0.00585	0.59267

L. bocagei Sado	*L. sclateri* Segura	0.00733	0.00968	0.75706
L. bocagei Sado	*L. graellsii* Ebro	0.01323	0.01481	0.89333
L. bocagei Sado	*L. guiraonis* Júcar	0.02233	0.03138	0.71153
L. bocagei Sado	*L. guiraonis* Mijares	0.01383	0.01493	0.92636
L. bocagei Sado	*H. haasi* Ebro	0.03817	0.03905	0.97766
L. comizo Guadiana	*L. steindachneri* Guadiana	-0.00006	0.00480	-0.01159
L. comizo Guadiana	*L. microcephalus* Guadiana	0.01115	0.01456	0.76551
L. comizo Guadiana	*L. sclateri* Guadiana	0.00160	0.00549	0.29124
L. comizo Guadiana	*L. sclateri* Segura	0.00549	0.00935	0.58744
L. comizo Guadiana	*L. graellsii* Ebro	0.01359	0.01668	0.81492
L. comizo Guadiana	*L. guiraonis* Júcar	0.02231	0.03287	0.67877
L. comizo Guadiana	*L. guiraonis* Mijares	0.01421	0.01681	0.84501
L. comizo Guadiana	*H. haasi* Ebro	0.03799	0.04037	0.94107
L. steindachneri Guadiana	*L. microcephalus* Guadiana	0.01093	0.01444	0.75703
L. steindachneri Guadiana	*L. sclateri* Guadiana	0.00092	0.00490	0.18742
L. steindachneri Guadiana	*L. sclateri* Segura	0.00451	0.00846	0.53301
L. steindachneri Guadiana	*L. graellsii* Ebro	0.01340	0.01658	0.80813
L. steindachneri Guadiana	*L. guiraonis* Júcar	0.02240	0.03306	0.67768
L. steindachneri Guadiana	*L. guiraonis* Mijares	0.01402	0.01672	0.83849
L. steindachneri Guadiana	*H. haasi* Ebro	0.03819	0.04067	0.93918
L. microcephalus Guadiana	*L. sclateri* Guadiana	0.01158	0.01413	0.81969
L. microcephalus Guadiana	*L. sclateri* Segura	0.01343	0.01594	0.84225
L. microcephalus Guadiana	*L. graellsii* Ebro	0.00173	0.00347	0.49803
L. microcephalus Guadiana	*L. guiraonis* Júcar	0.01737	0.02659	0.65328
L. microcephalus Guadiana	*L. guiraonis* Mijares	0.00194	0.00320	0.60563
L. microcephalus Guadiana	*H. haasi* Ebro	0.03618	0.03721	0.97216
L. sclateri Guadiana	*L. sclateri* Segura	0.00271	0.00570	0.47562
L. sclateri Guadiana	*L. graellsii* Ebro	0.01407	0.01629	0.86382
L. sclateri Guadiana	*L. guiraonis* Júcar	0.02372	0.03341	0.70991
L. sclateri Guadiana	*L. guiraonis* Mijares	0.01475	0.01648	0.89457
L. sclateri Guadiana	*H. haasi* Ebro	0.03976	0.04128	0.96339
L. sclateri Segura	*L. graellsii* Ebro	0.01550	0.01768	0.87636
L. sclateri Segura	*L. guiraonis* Júcar	0.02587	0.03553	0.72810
L. sclateri Segura	*L. guiraonis* Mijares	0.01657	0.01827	0.90667
L. sclateri Segura	*H. haasi* Ebro	0.04205	0.04353	0.96603
L. graellsii Ebro	*L. guiraonis* Júcar	0.01919	0.02808	0.68338
L. graellsii Ebro	*L. guiraonis* Mijares	0.00429	0.00522	0.82094
L. graellsii Ebro	*H. haasi* Ebro	0.03777	0.03848	0.98160
L. guiraonis Júcar	*L. guiraonis* Mijares	0.01917	0.02758	0.69515
L. guiraonis Júcar	*H. haasi* Ebro	0.00289	0.01107	0.26073
L. guiraonis Mijares	*H. haasi* Ebro	0.03808	0.03831	0.99407

D_a: relative average diveregence corrected for within species divergence; D_{xy}: absolute average divergence; F_{ST}: fixation index
