# Supplementary material for: Semi-permeable species boundaries in Iberian barbels (Barbus and Luciobarbus, Cyprinidae)
Source: BMC Evol Biol. 2015 Jun 12;15:111. doi: 10.1186/s12862-015-0392-3 (PMC4465174; doi:10.1186/s12862-015-0392-3)

Additional file 2 – Population polymorphism networks across loci

D_a: relative average divergence corrected for within species divergence

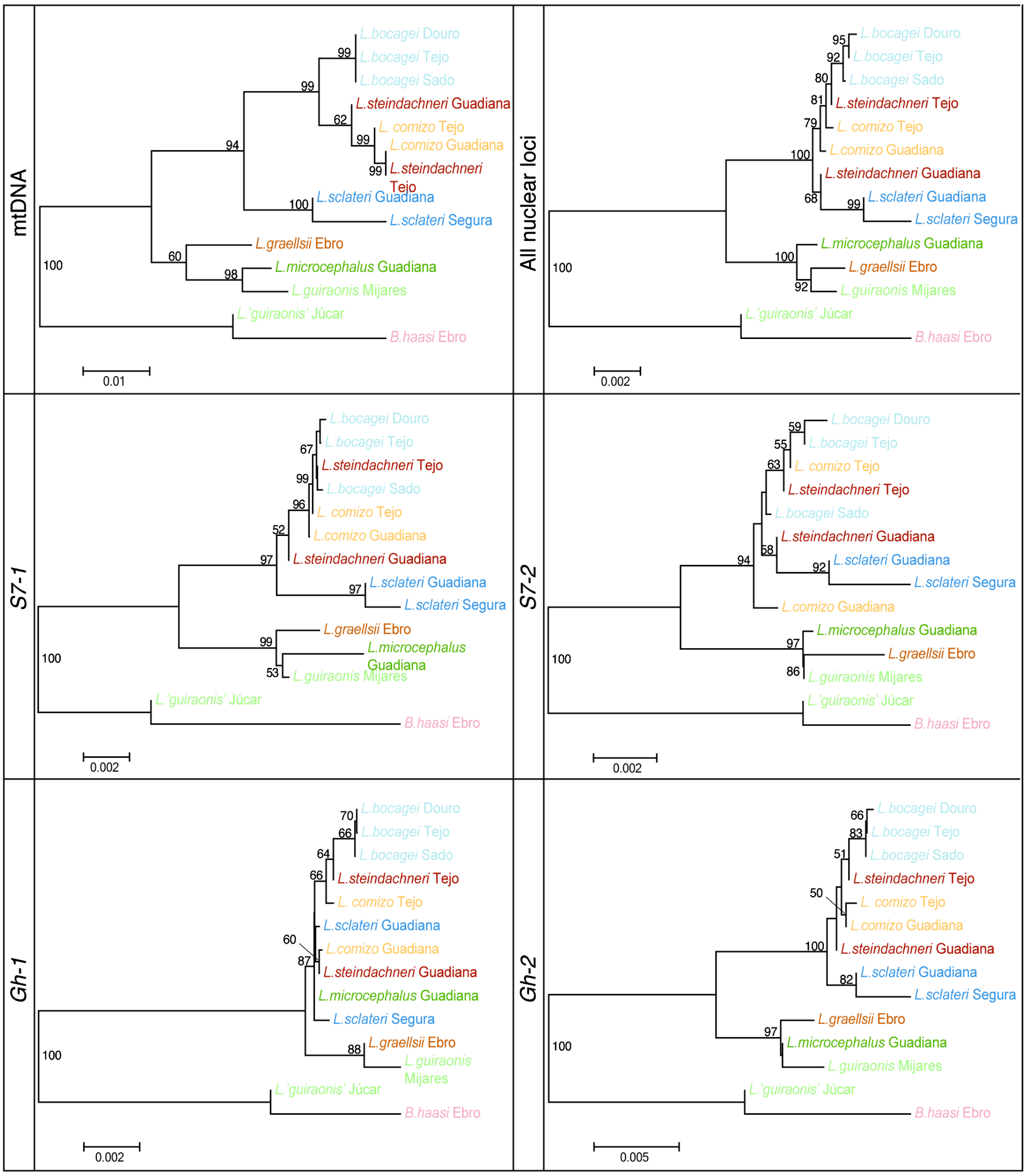

D_{xy}: absolute average divergence

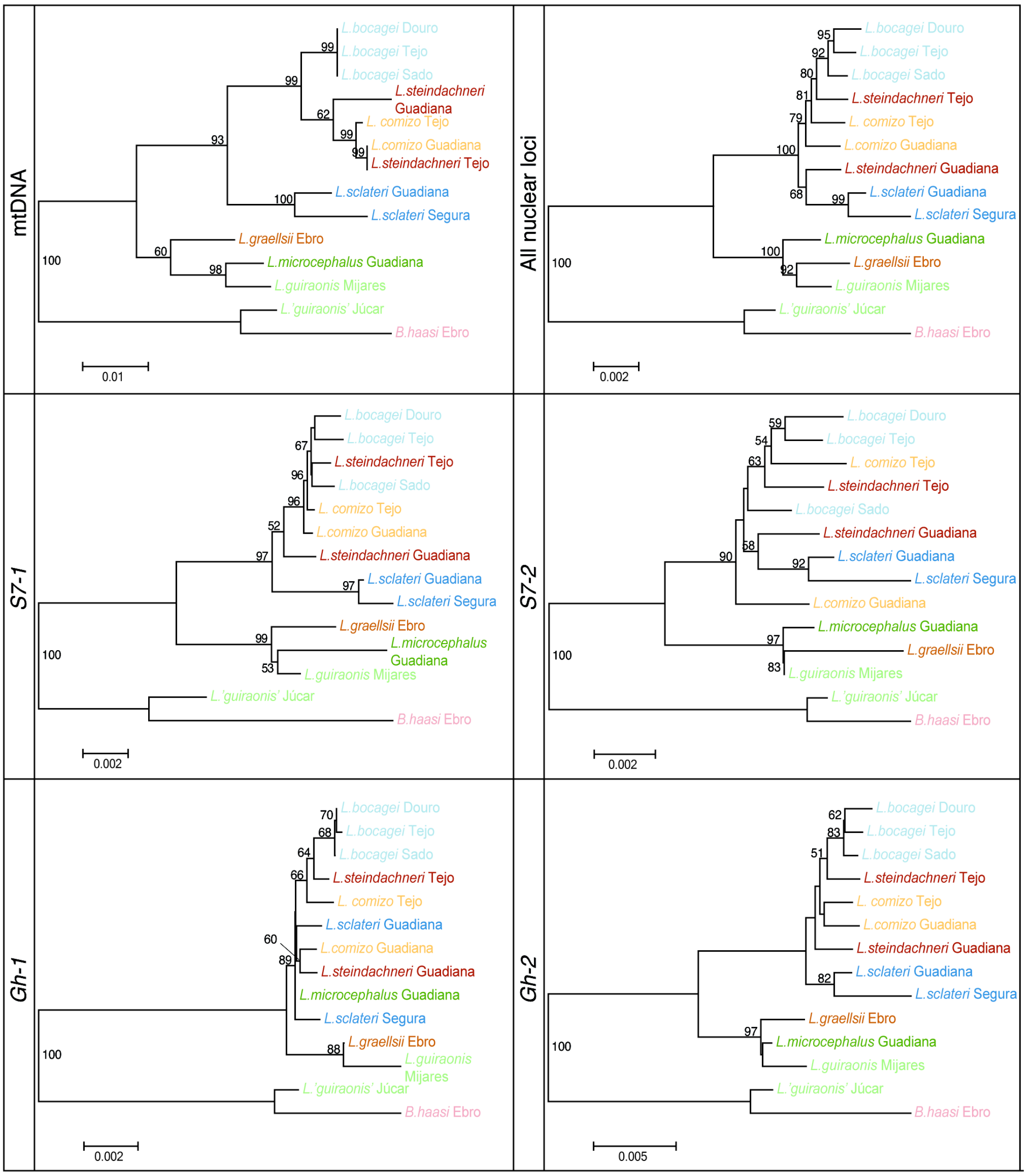

F_{ST}: fixation index

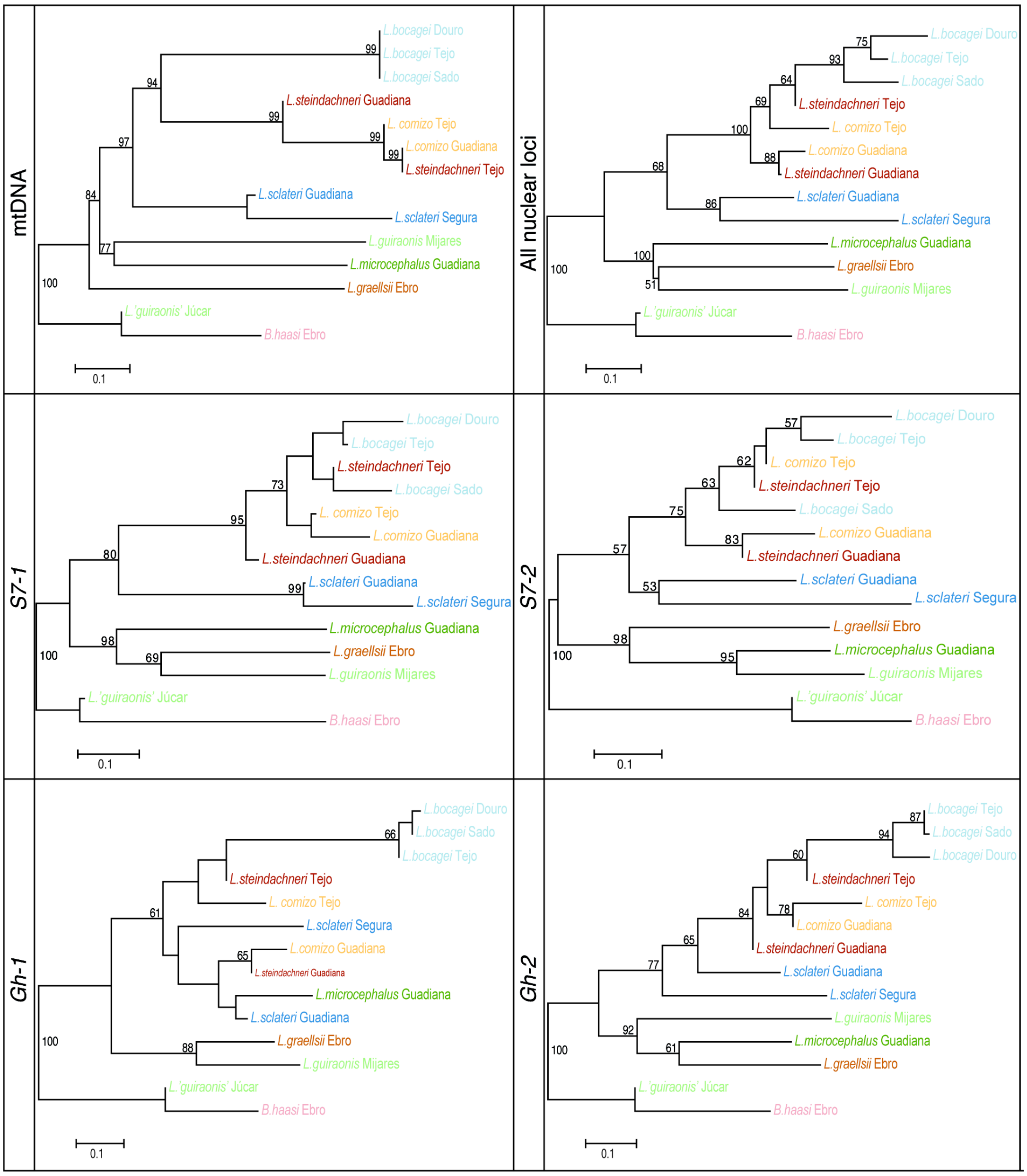

Supplement: Additional file 2: — Population polymorphisms networks across loci. [file 12862_2015_392_MOESM2_ESM.pdf]
