# Supplementary material for: Semi-permeable species boundaries in Iberian barbels (Barbus and Luciobarbus, Cyprinidae)
Source: BMC Evol Biol. 2015 Jun 12;15:111. doi: 10.1186/s12862-015-0392-3 (PMC4465174; doi:10.1186/s12862-015-0392-3)
Supplement: Additional file 3: — STRUCTURE analysis using different numbers of samples. [file 12862_2015_392_MOESM3_ESM.pdf]

Additional file 3 – STRUCTURE analysis using the complete dataset

Bayesian clustering of nuclear data using the highest likelihood value

Due to high multimodality of the nuclear dataset (i.e. large number of clusters), STRUCTURE sometimes converged to different solutions in independent replicates of each *K*, making determination of the best *K* challenging (a phenomenon already described in the literature and explained in the software's manual). Consequently, this behavior negatively affected Δ*K* calculations, leading to a severe underestimation of *K* following Evanno's method (*K*=2 using Δ*K* for the entire dataset). This happens because exceptionally good replicate runs, for exceptionally hard solutions (such as high *K*), are averaged with poor runs that converged to sub-optimal solutions [i.e. low LnP(D)] when calculating Δ*K*. Therefore, we discourage the use of Evanno's method in these situations and propose an alternative strategy to the split-and-reanalyze strategy to determine *K* presented in the main text. Our alternative strategy consists of running a high number of iterations per *K* (at least 40 replicates) to ensure that multiple modes are hit and that an optimal solution [i.e. highest possible LnP(D) per *K*] is hit one or a few times by the MCMC algorithm. While not averaging results across replicates, the run with the highest value of LnP(D) should indicate the best clustering solution for each *K*, and the run with the highest overall LnP(D) should indicate the *K*-value best supported by the data (i.e. the most biologically meaningful result). To illustrate this strategy with our data, we show four consecutive *K*-solutions (*K*=8 to *K*=11) where LnP(D) values plateau and look at the individual runs that have the highest LnP(D) values for each *K*. The run with the best LnP(D) for *K*=8 coincides with the second highest Δ*K*, which is sometimes used as a criterion when *K*=2 has the highest Δ*K* but a low LnP(D). In our case, from a biological standpoint, this solution corresponds to a sub-optimal value of *K* for which *L. guiraonis* and *B.*

haasi from different populations are grouped in one cluster. A few runs at higher *K*-values (*K*=9, *K*=10 and *K*=11) hit modes with higher LnP(D) and provide the most biologically sound solutions. *K*=9 allows the discrimination of all species included in the study, as well as allopatric populations of *L. comizo* and *L. sclateri*. *K*=10 and *K*=11 allow further recognition of population differentiation within *L. bocagei*. Overall, the latter is not only the best run for *K*=11, but also for all *K*-values, indicating that a possible maximum number of clusters has been identified. These results match those presented in the main text when analyzing partial datasets with the split-and-reanalyze strategy (Fig. 8). This new strategy presents the advantage, over consecutively splitting the dataset presented in the main text, of not excluding any of the genetic variants from partial datasets. Hence, it should present a better picture of how variation is shared across all species and to what extent populations are differentiated from one another. A clear example is the difference in the ability to detect genetic differentiation within *L. bocagei* between the split-and-reanalyze approach and the highest-likelihood-value approach we present here. We think this is a biologically sound result as other studies have found genetic differentiation between fish populations inhabiting the Sado and Tejo basins [1–3].

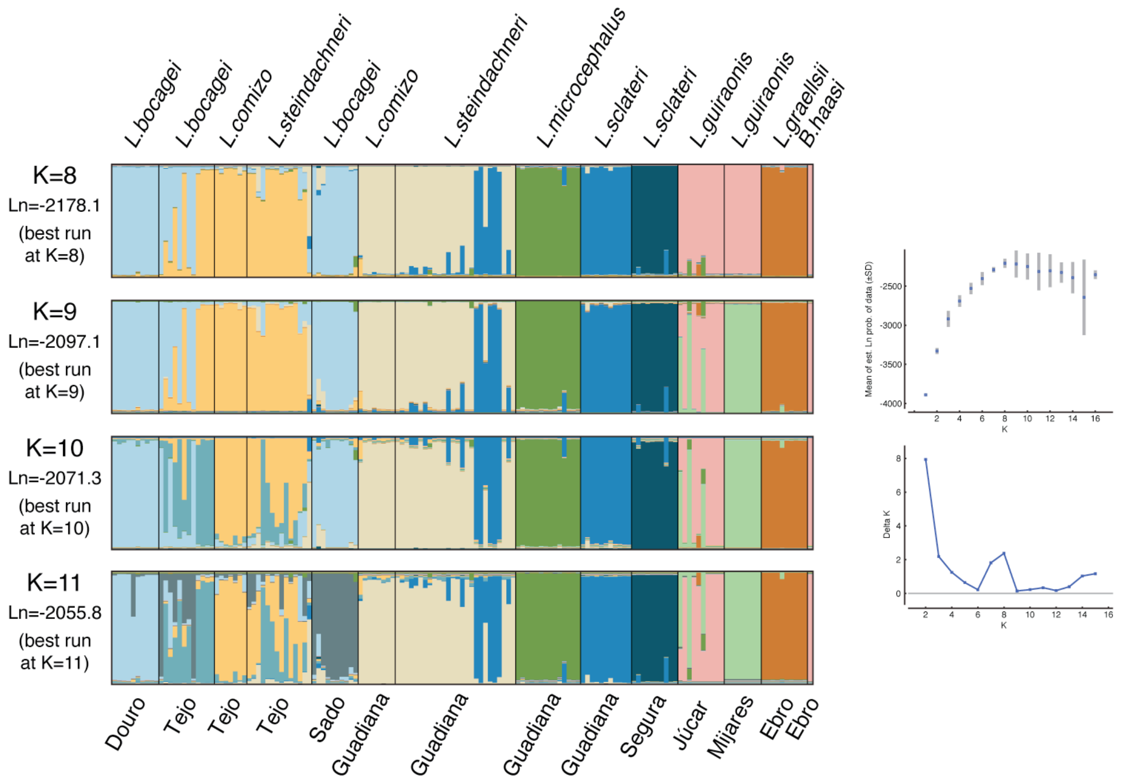

Figure 1 - Bayesian analysis of population structure.
Highest-likelihood-value approach implemented in STRUCTURE. Each panel shows the run with the highest LnP(D) for each *K* for a range of *K*-values where LnP(D) plateaus (*K*=8 to *K*=11). These analyses indicate that overall *K* = 11 populations is the most biologically meaningful genetic structuring of the entire nuclear dataset.

References

1. Cunha C, Coelho MM, Carmona JA, Doadrio I: **Phylogeographical insights into the origins of the *Squalius alburnoides* complex via multiple hybridization events**. *Mol Ecol* 2004, **13**:2807–17.

2. Sousa V, Penha F, Collares-Pereira MJ, Chikhi L, Coelho MM: **Genetic structure and signature of population decrease in the critically endangered freshwater cyprinid *Chondrostoma lusitanicum***. *Conserv Genet* 2007, **9**:791–805.

3. Sousa-Santos C, Collares-Pereira MJ, Almada V: **Reading the history of a hybrid fish complex from its molecular record.** *Mol Phylogenet Evol* 2007, **45**:981–96.
